# From paradox to target: IFN-β hijacks MEK signaling to drive a cell death-evading dormant phenotype in colorectal cancer

**DOI:** 10.1186/s13046-026-03768-6

**Published:** 2026-06-27

**Authors:** Yangyang Zhou, Haigang Geng, Yi Xu, Yanggang Hong, Bo Mei, Huanglei Wu, Xuanxuan Jin, Mengfan Ye, Yi Wang, Zan Shen, Zhigang Zheng, Zhenhua Zhu, Xiangchou Yang, Zizhen Zhang, Chunchao Zhu

**Affiliations:** 1 Department of Oncology, Shanghai Sixth People’s Hospital Affiliated to Shanghai Jiao Tong University School of Medicine, Shanghai, 200030 China; 2 Department of Gastrointestinal Surgery, Renji Hospital, Shanghai Jiao Tong University School of Medicine, Shanghai, China; 3 Department of Gastrointestinal Cancer Translational Research, Key Laboratory of Carcinogenesis and Translational Research (Ministry of Education), Peking University Cancer Hospital, Beijing, China; 4 State Key Laboratory of Systems Medicine for Cancer, Shanghai Cancer Institute, Renji Hospital, Shanghai Jiao Tong University School of Medicine, Shanghai, China; 5 The Second School of Medicine, Wenzhou Medical University, Wenzhou, Zhejiang China; 6 Department of Nuclear Medicine, Peking University Cancer Hospital & Institute, Key Laboratory of Carcinogenesis and Translational Research (Ministry of Education), Beijing Key Laboratory of Research, Investigation and Evaluation of Radiopharmaceuticals, NMPA Key Laboratory for Research and Evaluation of Radiopharmaceuticals (National Medical Products Administration), Beijing, 100142 China; 7 Department of Coloprotology, The Second Affiliated Hospital & Yuying Children’s Hospital of Wenzhou Medical University, Wenzhou, China; 8 Department of Urology, The Second Affiliated Hospital & Yuying Children’s Hospital of Wenzhou Medical University, Wenzhou, China; 9 The First School of Medicine, School of Information and Engineering, Wenzhou Medical University, Wenzhou, Zhejiang China; 10 Shanghai Cancer Institute, State Key Laboratory of Systems Medicine for Cancer, Renji Hospital, Shanghai Jiao Tong University School of Medicine, Shanghai, China; 11 Department of Pathophysiology, Key Laboratory of Cell Differentiation and Apoptosis of Chinese Ministry of Education, Shanghai Jiao Tong University School of Medicine, Shanghai, China; 12 Department of Hematology and Medical Oncology, The Second Affiliated Hospital & Yuying Children’s Hospital of Wenzhou Medical University, Wenzhou, China

**Keywords:** Tumor dormancy, Interferon-β, Therapy resistance, Colorectal cancer, MEK inhibition

## Abstract

**Background:**

The high clinical recurrence rate of colorectal cancer (CRC) is driven by the survival of residual tumor cells that evade therapy-induced death by entering a dormant state. While dormancy is a recognized mechanism of treatment resistance, the molecular drivers governing this "quiescent reservoir" and its associated vulnerabilities remain poorly characterized, limiting the development of strategies to eradicate these dormant seeds.

**Methods:**

We developed a COAD-specific Dormancy Score (CADS) derived from NMF analysis of ~ 69,000 single cells to quantify and identify a dormant subpopulation at single-cell resolution. Mechanistically, the IFN-β/cDC1 axis and its downstream MEK/ERK dependency were validated using a GFP-p27K- dormancy reporter system, spatial transcriptomics, and CRISPR/Cas9-mediated Ifnar1 knockdown. Finally, the synergistic efficacy of anti-PD-1 combined with MEK inhibition (Trametinib) was evaluated in orthotopic CRC mouse models.

**Results:**

The CADS effectively identified a distinct dormant subpopulation in CRC characterized by profound G0/G1 arrest, enhanced stemness, and multi-drug resistance. We uncovered a novel evasion mechanism mediated by the hijacking of IFN-β signaling. Conventionally recognized for its anti-proliferative roles, IFN-β signaling is exploited by surviving tumor cells to enter a deep quiescent state. This phenotype acts as a biological reservoir that fuels intratumoral heterogeneity and underpins the relapse of colorectal tumors by conferring resistance to conventional cytotoxic regimens. Effective anti-PD-1 therapy paradoxically enriches this dormant population via an enhanced IFN-β-conventional type 1 dendritic cell (cDC1) axis. Mechanistically, IFN-β–induced dormancy depends on MEK/ERK pathway activity, which sustains survival while suppressing apoptosis. This creates a synthetic lethal vulnerability: MEK inhibition (e.g., Trametinib) synergizes with IFN-β to re-sensitize dormant cells to apoptosis. Consequently, combining Trametinib with anti-PD-1 therapy overcomes this evasion mechanism, eliminates the dormant subpopulation, remodels the immune microenvironment, and shows strong synergistic efficacy in preclinical models.

**Conclusion:**

Our work redefines an immune–cell death paradox, revealing how tumors exploit IFN-β to evade therapy. We propose CADS as a translational biomarker for identifying tumors reliant on this pathway and validate a mechanism-based combination therapy that selectively targets dormancy-associated death resistance, offering a promising strategy to improve CRC outcomes.

**Supplementary Information:**

The online version contains supplementary material available at https://doi.org/10.1186/s13046-026-03768-6.

## Background

Phenotypic plasticity and drug-resistance persistence are considered core mechanisms driving resistance to both chemotherapy and immune checkpoint inhibitors (ICIs) in cancer [1, 2]. As an emerging hallmark, epigenetic plasticity imparts highly differentiated tumor cells with the ability to dedifferentiate under specific therapeutic stress, leading to a cellular state reprogramming. This reprogramming allows the cells to reacquire or maintain malignant features, such as drug resistance persistence, thereby enhancing therapeutic failure [3]. This plasticity-mediated cellular state reprogramming [4], particularly the shift toward a non-proliferative, high-survival state, represents a pivotal challenge in contemporary oncology research. This link between plasticity and therapeutic resistance has been substantiated across various tumor types [5, 6], being particularly pronounced in Colorectal Cancer (CRC) [7, 8]. As a gastrointestinal malignancy ranking among the global leaders in incidence and mortality, clinical treatment for CRC is persistently challenged by initial adequate response but unsatisfactory long-term outcomes, resulting in persistently poor five-year survival rates [9]. A key reason for this dilemma lies in the fact that therapeutic agents often induce a minority of tumor cells to enter a state of dormancy [10–12] within the tumor microenvironment (TME). This dormant phenotype is a specialized form of drug-tolerant persister (DTP) state, characterized by cell cycle arrest, reduced metabolic rate, and inherent resistance to conventional cytotoxic drugs [13]. It represents a critical survival strategy implemented by malignant cells through phenotypic plasticity.

An increasing body of evidence indicates that tumor cells can "hijack" immune signals within the TME through the aberrant expression of regulatory factors or the activation of compensatory pathways, allowing them to evade immune clearance [14]. This phenomenon of "immune signal hijacking" reveals an unexpected, bidirectional, and malignant adaptive interaction between tumor cells and immune cells [15]. For instance, studies in hepatocellular carcinoma (HCC) models showed that tumor-secreted erythropoietin (EPO) interacts with its homologous receptor (EPOR) on tumor-associated macrophages (TAMs), thereby shaping an immunosuppressive, or "cold," TME [16]. In head and neck squamous cell carcinoma (HNSCC), cancer cells, under immune pressure, activate the ATF4-SLIT2-CGRP axis to stimulate nociceptive neurons innervating tumor-draining lymph nodes (TDLNs), remodeling the TDLNs into an immunosuppressive state [17]. Furthermore, breast cancer cells can hijack iron-rich macrophages to acquire iron and mimic erythrocyte characteristics, which subsequently dampens the efficacy of immune checkpoint blockade (ICB), enabling cancer cells to sustain proliferation and survival in the hypoxic bone marrow environment [18]. Intriguingly, Type I Interferons (IFN-I), particularly IFN-β, are classically viewed as crucial signaling molecules secreted by immune cells such as dendritic cells (DCs) to activate anti-tumor immune responses [19]. IFN-β is considered an essential component of tumor immunotherapy, functioning by modulating the TME and enhancing T cell effector functions [19]. However, the malignant adaptability of tumor cells often transcends classical immunological models. Here, we propose a paradoxical hypothesis: in the stringent selective environment of therapeutic pressure (such as chemotherapy or ICIs), DC-mediated IFN-β signaling might be captured by a minority of malignant tumor cells [20], and paradoxically "repurposed" via its downstream pathways. This process potentially induces a subset of malignant cells to enter an adaptive dormant phenotype characterized by high survival rate, thereby achieving “stem cell-like” phenotype and immune evasion. If substantiated, this IFN-β-driven dormancy mechanism would fundamentally alter our understanding of the dual role of IFN-β in the TME and provide an explanation for the survival origin of DTP cells in post-treatment recurrent tumors. A high-resolution quantification standard and a systematic mechanistic dissection of this malignant dormant phenotype-which is "reverse-induced" by an immune signal-remain largely absent in the field of immunotherapy, a gap this study primarily addresses. Accordingly, this study is dedicated to developing high-resolution tools and systematically dissecting this immune-signal reverse-driven malignant dormancy mechanism. Based on single-cell transcriptomic analysis, we developed the COAD-specific Dormancy Score (CADS), a precise, single-cell level metric for quantifying the malignant dormant phenotype in CRC. Unlike traditional dormancy markers based solely on cell cycle arrest, CADS integrates key gene features related to adaptive survival and immune evasion, providing a more comprehensive and high-resolution characterization of this malignant adaptive phenotype. We definitively established that the high-dormancy phenotype is a core malignant feature of CRC, exhibiting a strong positive correlation with tumor recurrence and intratumor heterogeneity, thereby revealing the dormant state as a highly aggressive survival strategy for tumor cells.

This study systematically elucidates and validates the intrinsic logic by which the CADS-captured dormant phenotype orchestrates therapeutic resistance. At the cellular level, the high-dormancy phenotype enables cells to activate specialized survival programs, thereby acquiring direct multi-drug resistance to broad-spectrum chemotherapeutics. Intriguingly, this dormant state simultaneously unmasks a discrete therapeutic vulnerability: pharmacological inhibition of the MEK signaling pathway synergizes potently with IFN-β signaling, bypassing established cellular survival defenses to trigger apoptosis. Further translational investigations demonstrate that integrating this combination regimen with anti-PD-1 therapy yields a significant dual anti-tumor effect.

## Methods

### Public data acquisition and preprocessing

#### TCGA pan-cancer cohort data

RNA sequencing (RNA-seq) transcriptome data (FPKM values), corresponding clinical follow-up information (including Overall Survival, OS, and Recurrence-Free Survival, RFS), somatic tumor mutation burden (TMB, or Mutation Load), and Copy Number Variation (CNV) data for the pan-cancer cohort were systematically retrieved from The Cancer Genome Atlas (TCGA) database TCGAbiolinks (v2.25.3) R package [21].

#### Cell line and drug sensitivity databases

Gene expression profiles covering 1,393 cell lines were obtained from the Cancer Cell Line Encyclopedia (CCLE) database [22] for subsequent Dormancy score (DS) calculation and biological pathway enrichment analysis. Drug sensitivity data were integrated from the Genomics of Drug Sensitivity in Cancer (GDSC) [23], Cancer Therapeutics Response Portal (CTRP), and PRISM databases. The area under the drug dose–response curve (AUC) was used as the primary metric to assess differences in sensitivity to conventional chemotherapeutic agents between high-DS and low-DS cell lines.

#### External validation

Multiple independent external gene expression datasets (GSE15622 [24], GSE70233 [25], GSE8465 [26], GSE19860 [27], GSE35935, GSE14764 [28], GSE14615 [29], GSE19293 [30]) were collected from the Gene Expression Omnibus (GEO) database to validate the prognostic predictive capability of the DS, quantified by the Area Under the Receiver Operating Characteristic (ROC) curve. Single-cell RNA sequencing (scRNA-seq) datasets for CRC (GSE178341 [31], LEE cohort, GSE166555 [32]) were also downloaded from the GEO database. 2015_Van Allen [33], 2017_Nathanson [34], 2017_Riaz [35], 2018_Auslander [36], 2020_Cho [37], 2019_Zhao [38] were collected from the GEO database to evaluating the efficacy of CADS or DS in predicting chemotherapy or immunotherapy response.

### Single-cell RNA sequencing (scRNA-seq)

#### Data acquisition and preprocessing

Three publicly available CRC scRNA-seq datasets (GSE178341, GSE166555, LEE cohort) were integrated and analyzed to construct a high-heterogeneity TME cell atlas. Raw count matrices were processed using the Seurat package (version 4.4.1) [39] in the R programming environment.

#### Strict quality control (QC) was applied to each dataset

Cells with the number of detected genes (nFeature_RNA) between 200 and 5,500 (adjusted based on the distribution of each dataset) were retained. Cells with Unique Molecular Identifier (UMI) counts greater than 500 were retained. Cells with mitochondrial gene expression percentage (calculated as the percentage of mitochondrial gene counts relative to total UMI counts) below 10% were excluded to filter out low-quality or dying cells.

Filtered data were normalized using the NormalizeData function in the Seurat package, employing the LogNormalize method, followed by a natural logarithm transformation. Data were projected onto a two-dimensional space for visualization using the UMAP (Uniform Manifold Approximation and Projection) algorithm based on Harmony embeddings for batch effect correction [40].

### Construction of the Dormancy Score 

Gene sets were integrated from two independent studies (Ren [41] laboratory: Ren_Dormant_UP, Ren_Reactive_UP and Weng [42] laboratory: Weng_Dormant_UP, Weng_Reactive_UP). Genes commonly upregulated in dormant cells (Dormant Gene Set) and commonly upregulated in reactive/proliferating cells (Reactive Gene Set) across both sources were identified. To ensure maximum data transparency and reproducibility, the comprehensive lists of genes defining the 'dormant_up' and 'reactive_up' subpopulations are fully documented in Supplementary Table S1. The single-sample Gene Set Enrichment Analysis (ssGSEA) [43] method was utilized to independently calculate an enrichment score for each gene set within every tumor sample.

1. Dormant Gene Set Enrichment Score (ES_1): Calculated using the combined gene set previously defined as upregulated in dormant cells.
2. Reactive Gene Set Enrichment Score (ES_2): Calculated using the combined gene set previously defined as upregulated in reactive cells.

The DS was then computed as the difference between the two enrichment scores:

- Dormancy Score = ES_1—ES_2

A high DS value indicates that the expression of dormancy-associated genes is higher than that of reactivation-associated genes in the sample, suggesting a tendency toward a deeper quiescent state. Conversely, a low DS value suggests a more active, proliferative/reactive state.

### Association analysis of dormancy score with clinicopathological and genomic features

#### Tissue type and recurrence

The DS was compared between tumor tissues and adjacent normal tissues, as well as between recurrent and non-recurrent samples within the TCGA pan-cancer cohort.

#### Genomic features

The correlation between DS and Tumor Mutation Burden (TMB) for TCGA pan-cancer samples was assessed using Pearson correlation analysis and further validated by plotting a scatter diagram in the CRC cohort. The correlation between DS and Copy Number Variation (CNV) score was similarly assessed using Pearson correlation analysis. Further sub-analyses investigated the correlation between DS and CNV at different resolution levels (focal, chromosomal arm, and whole chromosome).

#### Immunotherapy prediction

The TIDE (Tumor Immune Dysfunction and Exclusion) algorithm was used to predict immunotherapy efficacy scores (TIDE Score) in TCGA or independent cohorts. The correlation between the calculated DS and the TIDE Score was then analyzed [44, 45].

#### Dormancy score and chemosensitivity analysis

Cell line drug sensitivity data from the GDSC database were utilized. Cell lines were dichotomized into high-DS and low-DS groups based on the median DS value. The sensitivity difference between the two groups to 14 commonly used chemotherapeutic agents (including Oxaliplatin, Fluorouracil, Capecitabine, and Docetaxel) was compared. The AUC value of the drug dose–response curve served as the sensitivity indicator, where a smaller AUC indicates higher sensitivity.

#### Single-cell Copy Number Alteration (CNA) analysis

For scRNA-seq datasets (GSE178341), the InferCNV or analogous methods were employed to infer chromosome-level Copy Number Alterations (CNA) status for individual cells based on their RNA expression profiles. Cells exhibiting extensive aneuploidy and specific chromosomal alterations inferred from the CNA pattern were identified as Malignant Epithelial Cells. Conversely, cells with an average log_2 ratio close to 0 were defined as non-malignant or reference normal tissue cells.

#### CRISPR dependency score analysis

Cancer cell line gene CRISPR dependency scores were retrieved from the publicly available DepMap database [46]. The distribution of CRISPR dependency scores for defined Dormancy genes was compared against that of Random genes. Density plots and boxplots were used for visualization to assess whether dormancy genes are less or more likely to be essential for the survival of cancer cell lines (a lower dependency score indicates higher essentiality).

#### Pathway enrichment analysis

Gene Set Enrichment Analysis (GSEA) or Correlation Analysis was performed to assess the association between the DS and various biological pathways (e.g., cell activity, immune response, stemness, and dormancy-related pathways). The Regression coefficient and adjusted *P*-value between the DS and the activity score of each pathway were calculated. Results were presented for both the pan-cancer cohort and specific cancer types.

### Identification of COAD-specific dormancy features and prognostic association

#### Data and program identification

The GSE178341 dataset, generated using the 10X Genomics platform, provided single-cell transcriptome data for approximately 69,000 malignant epithelial cells. Non-Negative Matrix Factorization (NMF) was applied to decompose the complex expression data into multiple gene expression programmes, capturing cellular functional state heterogeneity [47].

#### Meta-programme definition

The identified gene expression programmes were subjected to correlation analysis against published pan-cancer dormancy signatures and various other biological signatures, resulting in a correlation coefficient matrix. Clustering analysis was performed on this matrix to define a set of more macroscopic and functionally distinct Meta-programmes. This analysis aimed to precisely delineate the tumor cell dormancy-associated Meta-programme(s) from the scRNA-seq data and validate the association of the core gene sets derived from these programmes with malignant tumor features.

#### Assessment of transcriptomic Intratumoral Heterogeneity (ITH)

To evaluate the degree of intratumoral transcriptomic divergence, we employed a distance-based metric applied to malignant cell populations. Following normalization and log-transformation of the raw count matrix, we identified the top 2,000 highly variable genes (HVGs) to capture the principal axes of variation. Malignant cells from each clinical specimen were projected into a high-dimensional feature space-refined by Principal Component Analysis-where pairwise Euclidean distances were systematically computed between all intra-sample cell pairs. The ITH intensity for each patient was quantified as the mean of these reciprocal distances. Under this framework, elevated Euclidean coefficients serve as a proxy for heightened transcriptional instability and increased cellular plasticity within the tumor architecture.

#### Plasmid sources and construction

Modification of the pCDH-EF1 Vector: The original pCDH-EF1-mVenus-p27K^−^ plasmid (Addgene, #176,651) was modified [48]. The mVenus (yellow fluorescent protein) coding sequence was replaced with the GFP gene via PCR to achieve green fluorescence. The final construct encoded the GFP-p27K^−^ green fusion protein. Acquisition of pLenti-CBh Vector: The pLenti-CBh-3xFLAG-Luc2-tCMV-tdTomato-F2A-BSR-WPRE vector (#H9912) was purchased from OBiO Technology (Shanghai) Company.

##### Ifnar1 knockdown validation

Lysates from sgCtrl and sgIfnar1 cells were collected. Western Blotting was performed using an anti-Ifnar1 antibody (64 kDa) to detect protein expression levels, with anti-β-actin (45 kDa) serving as the internal loading control, to confirm the Ifnar1 knockdown efficiency. The sgRNA targeting the mouse Ifnar1 gene was cloned into the lentiCRISPR v2 vector. The targeting sequences were as follows: 5’- TTCAGCAGAATATCGAACGTAGG’. Lentiviral particles were produced in HEK293T cells and used to infect MC38 cells, followed by selection with 2 μg/ml puromycin for 7 days.

#### CADS cutoff robustness analysis, CSC, and proliferation scoring

To evaluate the mathematical stability of the CADS, malignant cells were stratified into CADS-high and CADS-low subgroups utilizing four distinct quantile thresholds: the top vs. bottom 1%, 5%, 25%, and 50%. The Cancer Stem Cell scores and Proliferation scores for individual malignant cells were calculated using the AddModuleScore function in Seurat, based on validated gene sets obtained from the Molecular Signatures Database (MSigDB).

#### Establishment and culture of patient-derived organoids (PDOs)

Human colorectal cancer tissues were obtained from patients undergoing surgical resection at Renji Hospital, with written informed consent from all participants and approval from the Institutional Review Board (Approval No.KY2024-188-C). Patient-derived organoids (PDOs) were established as previously described [49]. Two representative lines, designated as PDO-1 (low baseline CADS) and PDO-2 (high baseline CADS), were expanded and frozen at early passages for subsequent functional validation.

#### Bulk RNA sequencing of PDOs

Total RNA was extracted from PDO-1 and PDO-2 using TRIzol reagent (Invitrogen). RNA integrity was validated using an Agilent 2100 Bioanalyzer. mRNA libraries were prepared using the TruSeq RNA Sample Preparation Kit (Illumina) and sequenced on an Illumina NovaSeq 6000 instrument to generate 150-bp paired-end reads. Differentially expressed genes between the two groups were evaluated, focusing on established dormancy markers (NR2F1, CDKN2A) and proliferation markers (CCND1, PCNA, MKI67, TOP2A).

#### EdU incorporation, chemoresistance, and apoptosis assays in PDOs

For the cell proliferation assay, PDOs were incubated with 10 μM 5-ethynyl-2'-deoxyuridine (EdU; Beyotime, Cat# C0075S) for 4 h at 37 °C. Organoids were then fixed with 4% paraformaldehyde, permeabilized with 0.5% Triton X-100, and stained using the Click-iT EdU Alexa Fluor 488 Imaging Kit. The percentage of EdU-positive cells was quantified by confocal microscopy (Leica SP8).

To evaluate chemotherapeutic resistance, PDOs were seeded into 96-well plates embedded in Matrigel and challenged with vehicle (PBS) or a clinically relevant dose of FOLFOX regimen for 72 h.

#### In vivo Extreme Limiting Dilution Assay (ELDA) and tumor growth kinetics

To evaluate tumor-initiating cell (TIC) frequency, single-cell suspensions derived from mechanically disrupted PDO-1 and PDO-2 were mixed with Matrigel (1:1 v/v) and subcutaneously injected into the right flanks of 5-week-old female BALB/c nude mice at graded cell doses: 4 × 10^2^, 2 × 10^3^, 1 × 10^4^ cells per mouse (*n* = 5 mice per group). For therapeutic challenge groups, mice were intraperitoneally treated with FOLFOX (5-FU: 30 mg/kg, Oxaliplatin: 5 mg/kg, once weekly) or vehicle starting one week post-injection.

Tumor formation was monitored weekly for up to 6 weeks. Tumor dimensions were measured using digital calipers, and tumor volume (V) was computed using the formula: V = Length × Width^2^/2.

The TIC frequency and statistical significance were calculated via Extreme Limiting Dilution Analysis (ELDA) using the online software tool (http://bioinf.wehi.edu.au/software/elda/).

### Animal models

#### Subcutaneous tumor model and drug intervention

Cell Culture and Gene Knockdown: MC38 CRC cells were gene-knocked down for Ifnar1 using the CRISPR/Cas9 system in vitro to generate sgIfnar1 cell lines. sgCtrl (negative control) cells were also prepared. Animal Model: 5 × 10^5^ sgCtrl or sgIfnar1 MC38 cells were subcutaneously injected into the flanks of C57BL/6 mice to establish the subcutaneous tumor model. Grouping and Treatment: Mice were randomly divided into four groups: sgCtrl, sgIfnar1, sgCtrl + anti-PD1, and sgIfnar1 + anti-PD1. anti-PD1 antibody or control IgG was administered via intraperitoneal (i.p.) injection on day 6, 10, 14, and 18. Tumor Monitoring: Tumor length and width were measured every 5 days, and tumor volume (mm^3^) was calculated using the formula (Length × Width^2^/2) to evaluate the combined efficacy of Ifnar1 knockdown and anti-PD1 treatment.

#### Orthotopic tumor model construction and treatment

Cell Preparation: 5 × 10^5^ luciferase-expressing MC38 CRC cells (MC38-Luc) were used for orthotopic tumor model construction. For orthotopic implantation, mice were anesthetized with 2% isoflurane and subjected to a 1-cm midline laparotomy to expose the distal colon. Under a dissecting microscope to ensure anatomical precision, cells were suspended in a 30 μL mixture of PBS and Matrigel (1:1 ratio) containing 0.1% Trypan Blue. The cell suspension was injected into the colonic wall using a 34-gauge Hamilton syringe at a shallow angle (< 15°). The formation of a distinct blue sub-serosal bleb without intraperitoneal leakage confirmed successful intramural delivery. Following the procedure, the colon was gently returned to the abdominal cavity, and the abdominal wall was sutured [50]. Grouping and Treatment: Mice were randomly divided into four groups: Control, Trametinib monotherapy, anti-PD1 monotherapy, and Combination therapy. Trametinib (0.25 mg/kg daily) was administered via oral gavage, and anti-PD1 (3 mg/kg every three days) via i.p. injection.

#### Combined therapy in subcutaneous allograft model (MC38)

MC38 cells were subcutaneously inoculated into C57BL/6 mice. Mice were randomly grouped into Control, Trametinib (0.25 mg/kg daily, oral gavage), anti-PD1 (3 mg/kg every three days, i.p. injection) monotherapies, and Combination therapy groups. Tumor Volume: Tumor size was measured using a digital caliper on Day 1, 4, 8, 11, 14. The tumor volume (V) was calculated according to the formula: V = length × width^2^/2, where length and width represent the longest and shortest diameters of the tumor, respectively [51, 52]. Tumor Weight: At the experimental endpoint (Day 14), mice were euthanized, and tumor weights (mg) were recorded. Body Weight Monitoring: Mouse body weight (g) was monitored throughout the treatment period to assess drug toxicity. All animal experiments were carried out in compliance with the ARRIVE guidelines and the Guide for the Care and Use of Laboratory Animals [53]. The study protocol was approved by the Ethics Committee for Animal Experimentation of Shanghai Yishang Biotechnology Co., Ltd. (Protocol ID:IACUC-2025-NI-054). Every effort was made to alleviate animal distress, including the use of appropriate anesthesia and monitoring during surgical and interventional procedures. 

#### Tumor growth monitoring (bioluminescence imaging)

Tumor growth dynamics were monitored using the In Vivo Imaging System (IVIS), a non-invasive in vivo imaging system. At specific time points (D7, D12, D17, D22, D27), mice were subjected to bioluminescence imaging after intraperitoneal injection of D-Luciferin substrate. Tumor growth kinetics were plotted based on the bioluminescence intensity (Log2 BLI (photons/s)) to evaluate the therapeutic efficacy. At the experimental endpoint, tumor tissues were collected for imaging and subsequent analysis.

#### Cell isolation and flow cytometry analysis

For the in vivo tracking of dormancy dynamics, MC38 colorectal cancer cells stably transfected with the dormancy reporter system were injected into mice to form tumors, followed by anti-PD-1 treatment. Upon tumor harvesting and tissue dissociation, total tumor-infiltrating immune cells were first enriched by isolating CD45^+^ cells via magnetic-activated cell sorting (MACS) using CD45 MicroBeads (Miltenyi Biotec) according to the manufacturer's protocol. Subsequently, the isolated cellular fractions were subjected to multi-color flow cytometry profiling and single-cell sequencing preparation. Flow cytometry data acquisition and high-resolution cell sorting were performed on a BD LSRFortessa X-20 flow cytometer (BD Biosciences) equipped with FACSDiva software. Gating strategies were established using single-stain and fluorescence-minus-one (FMO) controls to ensure sorting purity.

### Flow cytometry analysis

#### Dendritic cell subset analysis

Cells were stained with anti-MHC-II and anti-CD11c antibodies. The MHC-II^+^ CD11c^+^ population was identified, and the cDC1 subset was further distinguished using anti-XCR1 antibody. The proportion of cDC1 cells within the total DC population was quantified.

#### Tumor cell dormancy analysis

For MC38 cells expressing the dormancy reporter (GFP/p27K^−^), flow cytometry was used to analyze the percentage of GFP^+^ cells within the tdTomato^+^ malignant cell population. GFP^+^ cells represented the dormant state.

#### Apoptosis detection (flow cytometry)

To detect the apoptotic effect of combined therapy, HCT116 and SW620 cells were treated with DMSO, IFN-β (5 ng/ml) alone, or combined with Oxaliplatin (10 μM), Selumetinib (0.5 μM), or Trametinib (0.5 μM). Cells were harvested and stained using the Annexin V/Propidium Iodide (PI) double-staining kit. The percentage of Annexin V^+^ cells (PI^−^ and PI^+^) was quantified via flow cytometry (FACS) as a measure of apoptosis.

#### Tumor-infiltrating lymphocytes (TILs) analysis

Single-cell suspensions were prepared from orthotopic tumor tissues of mice in various treatment groups via mechanical and enzymatic digestion. TILs were enriched using density gradient centrifugation. Cells were stained with surface markers including CD45, CD3, CD8, and PD-1. Flow cytometry was used to quantify the proportion of CD8^+^ T cells within the total TILs (CD45^+^ or CD3^+^ cells), as well as the expression level of PD-1 on CD8^+^ T cells.

### Cell function assays

#### IFN-β quantification

An ELISA (Enzyme-Linked Immunosorbent Assay) kit was used to quantitatively analyze the concentration (pg/ml) of IFN-β in tumor tissue homogenates from Control and anti-PD1 treated groups.

#### Spatial gene set variation analysis

Spatial transcriptomics data were normalized using standard procedures, including library size normalization and log-transformation. Gene signatures for cDC1, Dormancy_CADS, and IFN-β signaling were obtained from published studies. Gene set variation analysis (GSVA) is a non-parametric, unsupervised method for estimating variation of gene set enrichment. We applied GSVA to normalized spatial expression data to calculate signature enrichment scores for cDC1, CADS, and IFN-β signaling pathways for each spatial spot [54]. The resulting GSVA scores were mapped back onto tissue sections to visualize the spatial distribution of immune-related functional states. 

#### Cell culture and drug treatment

Human colorectal carcinoma cell lines HCT116 and SW620 (both purchased from the ATCC bioresource center and regularly checked for Mycoplasma contamination) were maintained in DMEM medium supplemented with 10% Fetal Bovine Serum (FBS) and 1% Penicillin–Streptomycin at 37℃ in a humidified atmosphere with 5% CO_2_. In vitro drug treatments included: IFN-β at concentrations of 3 ng/ml and 5 ng/ml; Targeted/Chemotherapeutic drugs: Oxaliplatin (10 μM), Selumetinib (0.5 μM), and Trametinib (0.5 μM).

#### Proliferation assay (EDU/Hoechst staining)

Cells were treated with different concentrations of IFN-β for 48 h. 5-Ethynyl-2'-deoxyuridine (EDU) was added to label proliferating cells, and Hoechst was used to counterstain all cell nuclei. Images were captured using a fluorescence microscopy system. The cell proliferation rate was quantified by calculating the percentage of EDU^+^ cells relative to the total number of cells (Hoechst^+^) (Confluence of EDU/Hoechst (%)).

#### Senescence detection (β-galactosidase staining)

Senescence-associated β-galactosidase (SA-β-gal) staining kit was used according to the manufacturer's instructions to detect cellular senescence [55]. Following the specified treatment duration, cells were incubated with X-gal solution at pH 6.0 overnight. SA-β-gal positive (blue staining) cells were counted and quantified.

#### Cell metabolism analysis (glucose consumption)

HCT116 and SW620 cells were treated with various IFN-β concentrations for 48 h, and the cell culture supernatant was collected. A Glucose Uptake-Glo Assay kit was used to determine the residual glucose concentration in the supernatant. Cellular glucose consumption (mg/ml) was calculated by comparing the residual glucose concentration to the glucose content of fresh culture medium.

### Quantitative real-time PCR (RT-qPCR) validation

#### RNA extraction and reverse transcription

HCT116 and SW620 cells (Control, IFN-β, Trametinib, and Combination treatment groups) were collected. Total RNA was extracted using the TRIzol reagent, and cDNA was synthesized from the RNA using a commercial reverse transcription kit.

#### qPCR

qPCR was performed on a real-time fluorescence quantitative PCR instrument using the SYBR Green kit. The relative expression levels of the MAPK pathway downstream genes FOS, EGR1, and DUSP6 were calculated using the 2–∆∆Ct method, with β-actin serving as the endogenous reference gene. The primers used were:

- FOS-F: CCGGGGATAGCGCTCTTCTACT
- FOS-R: CCGCAGTCGGTGCGGAAGTC
- EGR1-F: CCACGCCGAACACTGACATT
- EGR1-R: GAGGGGTTAGCGAAGGCTG
- DUSP6-F: GAACTGTGGTGTCTTGGTACATT
- DUSP6-R: GTTCATCGACAGATTGAGCTTCT
- ACTB -F: CATGTACGTTGCTATCCAGGC
- ACTB -R: CTCCTTAATGTCACGCACGAT

#### Transcriptome and enrichment analysis

Tumor tissues were collected from the orthotopic CRC mouse model before and after anti-PD1 treatment, and from human CRC cells HCT116 after 48 h of IFN-β treatment, for RNA extraction and subsequent RNA-seq sequencing. GSEA was performed on the resulting transcriptome data. KEGG pathway enrichment analysis was conducted on the differentially expressed genes identified before and after anti-PD1 treatment. Key pathway changes were visualized using a Sankey diagram.

### Immunofluorescence (IF) staining and quantification

#### Tissue sectioning

Mouse tumor tissues were collected, fixed, dehydrated, paraffin-embedded, and sectioned.

#### Immunofluorescence

Sections underwent antigen retrieval and were incubated with primary antibodies against CD8 (T cell marker) and TNF-α (cytotoxic function marker), followed by incubation with corresponding fluorescently labeled secondary antibodies. DAPI was used to counterstain cell nuclei.

#### Microscopic imaging and data acquisition

Multi-color immunofluorescence (mIF) signals within CRC tumor sections were captured utilizing a high-resolution laser-scanning confocal microscope (Leica TCS SP8; Leica Microsystems, Wetzlar, Germany) equipped with Leica Application Suite X (LAS X) software. To ensure precise spatial resolution and spectral purity, images were acquired using a 63 ×/1.40 NA oil-immersion objective or a 20 ×/0.75 NA dry objective for extended field of views.

Sequential scanning mode was strictly applied to eliminate potential spectral bleeding and channel crosstalk among fluorophores. Laser intensity, pinhole diameter (set at 1.0 Airy unit), and detector gains were maintained identical across all experimental groups to ensure absolute comparative rigor. For cell-counting quantification, a minimum of five random, non-overlapping high-power fields (HPFs) per tissue slice were scanned. Signal intensity and positive cell percentages were processed and automatically quantified using ImageJ/FIJI software (version 1.53c; NIH, Bethesda, MD, USA).

##### Reagents, antibodies and equipment

Detailed information on all reagents and antibodies used in this study, including sources, manufacturers, and catalog numbers, is listed in Supplementary Table S2.

### Statistical analysis

All statistical analyses were performed using GraphPad Prism software (version 9) and the R language (version 4.4.1). Data from in vitro and in vivo functional experiments are presented as the Mean pm Standard Error of the Mean (SEM). Comparison Between Two Groups: Student's t-test was used. Comparison Among Multiple Groups: One-way ANOVA was used. Dynamic In Vivo Data: Tumor volume and body weight curves were analyzed using Two-way ANOVA. Correlation Analysis: Pearson correlation test was employed. Significance Criterion: *P* < 0.05 was considered statistically significant. Significance levels in figures are denoted as *, **, *** representing *P* < 0.05, *P* < 0.01, and *P* < 0.001, respectively; ns denotes no significant difference.

## Results

### Establishing an integrative framework to characterize tumor dormancy and therapeutic vulnerabilities

**Fig. 1:**
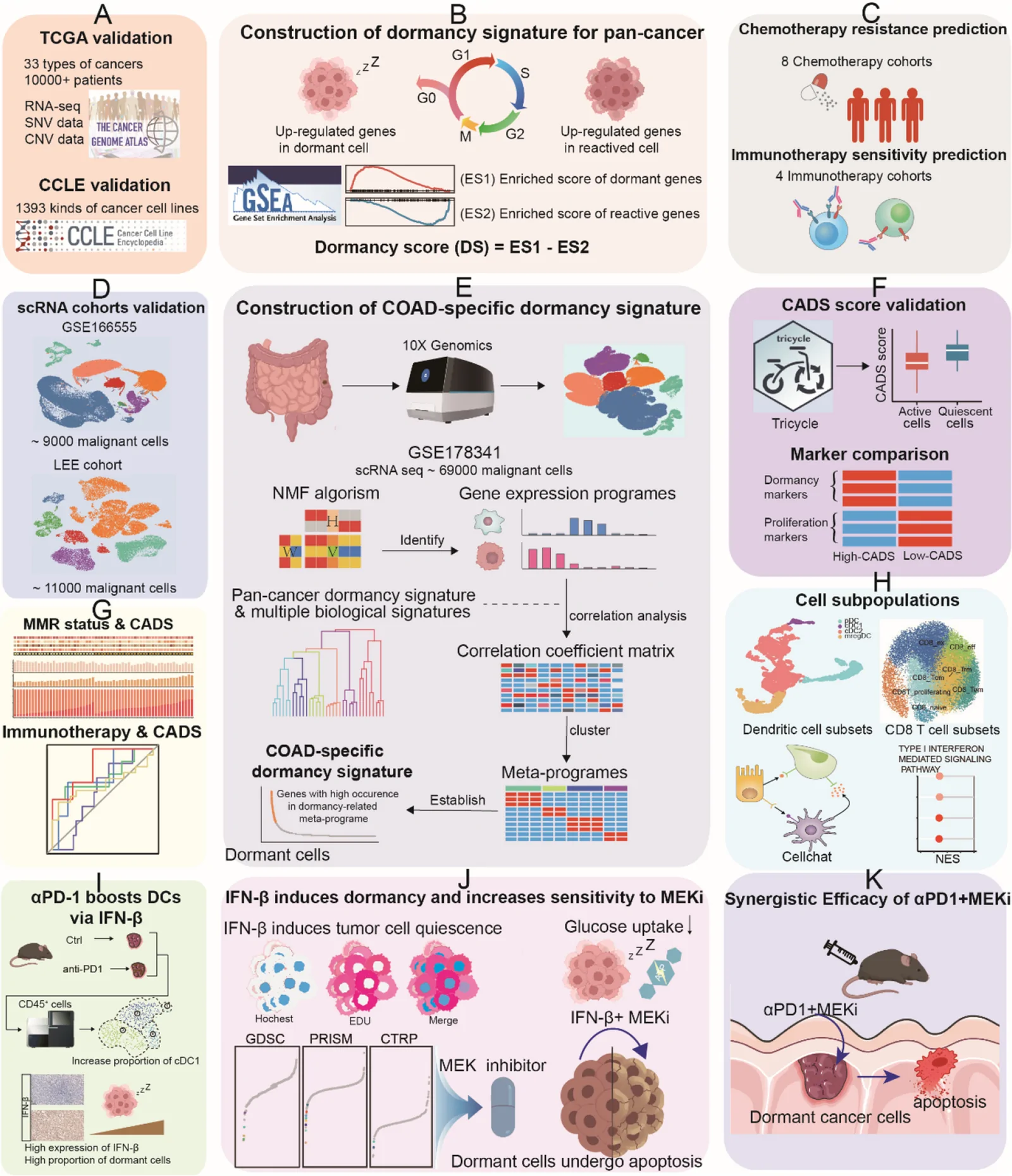
Establishing a Multi-stage Framework for Characterizing Tumor Dormancy and Therapeutic Vulnerability. **A**–**C** Construction and clinical predictive value of the pan-cancer dormancy signature: **A** Large-scale data integration using TCGA and CCLE (*n* = 1,393 cell lines) cohorts. **B** Schematic of the Dormancy Score (DS) derivation, defined as the difference between dormancy-associated (ES1) and activation-associated (ES2) enrichment scores via GSEA. **C** Performance of the DS in predicting chemotherapy resistance (8 cohorts) and immunotherapy sensitivity (4 cohorts). **D**–**H** Establishment and multi-dimensional characterization of the CADS: **D** Validation of dormancy programs in scRNA-seq cohorts (GSE166555 and Lee cohort). **E** Methodological pipeline for identifying COAD-specific dormancy meta-programs using NMF algorithms on around 69,000 malignant cells (GSE178341). **F** Functional benchmarking of CADS against cell-cycle phases (Tricycle algorithm) and established proliferation markers. **G** Clinical correlation of CADS with MMR status and immunotherapy outcomes. **H** Characterization of the dormant immune microenvironment, showing subset-specific infiltration (DC and CD8 + T cells) and intercellular communication via CellChat. **I**–**K** Mechanistic insights and combinatorial therapeutic evaluation: **I** Mechanism of Anti-PD1 therapy in modulating the IFN-β signaling axis to boost cDC1 recruitment. **J** Experimental evidence of IFN-β induced tumor cell quiescence and the identification of MEK inhibitors as a synthetic lethal intervention for dormant cells (verified via GDSC, PRISM, and CTRP). **K** Synergistic efficacy of combined anti-PD1 and MEK inhibition (Trametinib) in orthotopic CRC models, demonstrated by bioluminescence imaging and tumor growth kinetics. Abbreviations: TCGA, The Cancer Genome Atlas; CCLE, Cancer Cell Line Encyclopedia; GSEA, Gene Set Enrichment Analysis; ES, Enrichment Score; DS, Dormancy Score; scRNA-seq, single-cell RNA sequencing; COAD, colon adenocarcinoma; CADS, COAD-specific Dormancy Score; NMF, non-negative matrix factorization; MMR, mismatch repair; DC, dendritic cell; cDC1, conventional dendritic cell type 1; IFN-β, interferon-beta; GDSC, Genomics of Drug Sensitivity in Cancer; CTRP, Cancer Therapeutics Response Portal; anti-PD1, anti-programmed cell death protein 1

To systematically decipher the complex landscape of tumor dormancy and its clinical implications, we established a comprehensive research framework spanning computational discovery, clinical validation, and experimental mechanistic exploration (Fig. 1). The study was executed in three progressive phases:

- Phase I: Pan-cancer Signature Construction and Clinical Prediction (Fig. 1A–C): We first integrated large-scale transcriptomic and genomic data from TCGA (n > 10,000 patients) and CCLE (*n* = 1,393 cell lines) to define a robust pan-cancer dormancy signature (Fig. 1A). Using GSEA-based enrichment of dormancy-associated genes (ES1) and activation-associated genes (ES2), we developed a universal Dormancy Score (DS) (Fig. 1B) capable of predicting chemotherapy resistance and immunotherapy sensitivity across multiple clinical cohorts (Fig. 1C).
- Phase II: CADS Refinement and Multi-dimensional Validation (Fig. 1D–H): To address the heterogeneity of colon adenocarcinoma (COAD), we applied non-negative matrix factorization (NMF) to single-cell RNA-seq (scRNA-seq) data from approximately 69,000 malignant cells to refine the signature into the CADS (Fig. 1D-E). The CADS was rigorously benchmarked against tricyclic cell-cycle indexing and established proliferation markers to ensure its accuracy in identifying quiescent cell populations (Fig. 1F). Furthermore, we deciphered the dormant immune microenvironment, specifically identifying the enrichment of cDC1 and CD8^+^ T cell subsets in high-CADS regions (Fig. 1G-H).
- Phase III: Discovery of the IFN-Signal Paradox and Therapeutic Synergy (Fig. 1I–K): Building on these observations, we identified the IFN-Signal Paradox as a key survival mechanism. Experimental validation revealed that IFN-β induces tumor cell quiescence while simultaneously modulating the cDC1 axis (Fig. 1I-J). Crucially, this dormancy-related adaptive signaling established a "synthetic lethal" vulnerability to MEK inhibition, which was confirmed by the synergistic efficacy of combined anti-PD-1 and Trametinib therapy in orthotopic CRC models (Fig. 1K).

### Dormancy score development and prognostic value

**Fig. 2:**
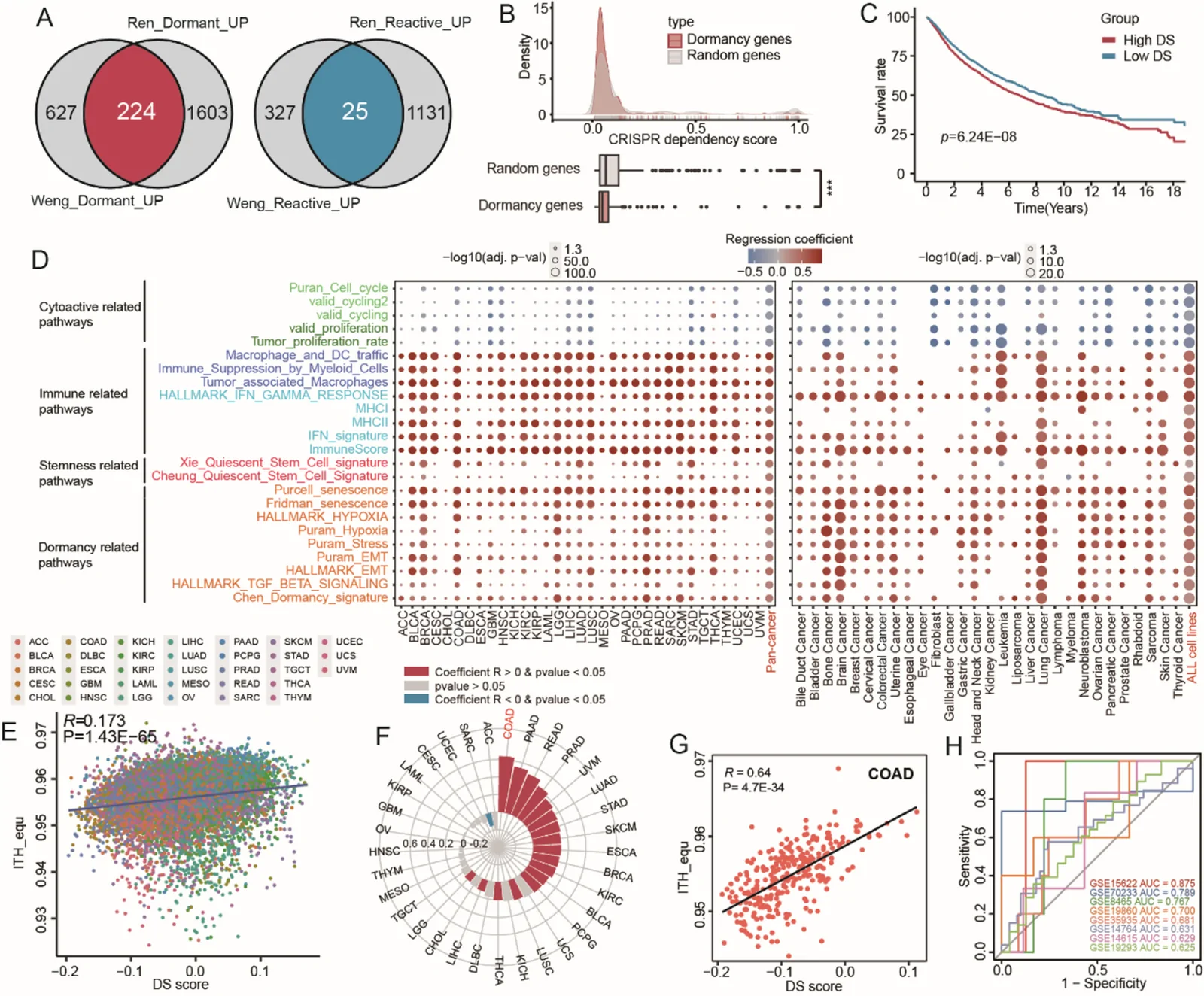
Cross-Cohort Validation and Functional Association of the Tumor Dormancy Signature. **A** Venn diagram illustrating the composition of the Dormancy Score (DS) gene set. The left panel shows the 224 intersecting genes between the quiescent upregulated genes reported by Ren et al. (Ren_Dormant_UP) and Weng et al. (Weng_Dormant_UP). The right panel displays the 25 intersecting genes between the reactive upregulated genes reported by Ren et al. (Ren_Reactive_UP) and Weng et al. (Weng_Reactive_UP). These shared genes collectively form the core set used for DS calculation. **B** Comparison of the CRISPR dependency scores between the core DS gene set (Dormancy genes) and randomly selected genes (Random genes). **C** Overall Survival (OS) curves for patients in the TCGA pan-cancer cohort stratified into high and low DS groups. The curve demonstrates that the high DS group exhibits significantly poorer long-term survival (*P* < 0.001, Log-rank (Mantel-Cox) test was applied to determine the statistical significance). **D** Correlation analysis of the DS with cytoactive, immune, stemness, and dormancy-related pathways across the TCGA pan-cancer cohort and the CCLE 1393 cancer cell line database. The *P*-value indicates the significance of the linear association (Pearson's). **E** Correlation of the DS with Intratumoral Heterogeneity (ITH_equ) across 33 TCGA cancer types. The *P*-value indicates the significance of the relationship (Pearson's). **F** Circular plot illustrating the correlation coefficient between the DS and ITH_equ across 33 TCGA cancer types. Color and shading intensity represent the strength and statistical significance of the correlation. The *P*-values were calculated for each cancer type to determine the significance of the association (Correlation coefficients and *P*-values). **G** Scatter plot highlighting the strongest positive correlation between the DS and ITH_equ observed in Colon Adenocarcinoma (TCGA-COAD) within the TCGA pan-cancer cohort (*P* < 0.001, Pearson's correlation coefficient was used to quantify the linear relationship). **H** Receiver Operating Characteristic (ROC) curves for predicting patient prognosis using the DS across various independent external validation datasets (GSE cohorts) with chemotherapy. The Area Under the Curve (AUC) values demonstrate the robust predictive capacity of the DS across multiple cohorts. The *P*-values are used to test the null hypothesis that AUC = 0.5. DS: Dormancy Score; UP: Upregulated; TCGA: The Cancer Genome Atlas; OS: Overall Survival; CCLE: Cancer Cell Line Encyclopedia; ITH_equ: Intratumoral Heterogeneity (quantified by evenness); COAD: Colon Adenocarcinoma; ROC: Receiver Operating Characteristic; AUC: Area Under the Curve

To quantify the dormant state of tumor cells, we integrated two previously published gene expression profiles associated with tumor cell quiescence (Dormancy) and proliferation (Reactive). Specifically, by taking the intersection of upregulated genes in the dormant state reported by Ren et al. and Weng et al. (224 genes) and the intersection of upregulated genes in the active state (25 genes), we defined a core Dormancy Gene Set (Fig. 2A). These genes were notably enriched in pathways such as cytokine production, MHC protein complex, and immune receptor activity (Fig. S1A). Intriguingly, we observed that the genes within this core set exhibited a significantly lower CRISPR-dependent score compared to a random gene set (Fig. 2B), suggesting that these genes are essential for cell survival.

Based on this core gene set, we developed a quantitative metric termed the Dormancy Score (DS). We then stratified patients in the TCGA pan-cancer cohort into a high DS group and a low DS group according to the median value. Consequently, we found that patients in the low DS group exhibited a significantly longer overall survival (Kaplan–Meier analysis, *P* < 0.001, Fig. 2C). These results collectively demonstrated that the DS score was a powerful prognostic biomarker. However, a pan-cancer analysis of TCGA tumor tissues and adjacent normal tissues revealed that tumor tissues, in the vast majority of TCGA cohorts, displayed a significantly lower DS compared to adjacent normal tissues (Fig. S1B). This evidence indirectly indicates that maintaining a quiescent state is antagonistic to the proliferation and invasive phenotype of tumors. Conversely, within the tumor tissues themselves, patients with recurrence showed a higher DS score than non-recurrent patients (*P* < 0.01), implying that cellular dormancy is not always a benign quiescent state but rather a highly adaptive survival strategy associated with poor prognosis (Fig. S1C).

### Biological and genomic characterization of the DS and its predictive value for treatment resistance

Further analysis indicated that across the TCGA pan-cancer and CCLE databases (1393 cell lines), the DS consistently showed a positive correlation with the enrichment scores of various stemness, hypoxia, and EMT-related pathways. Simultaneously, it exhibited a negative correlation with major cell proliferation-related pathways (Fig. 2D). Of note, the DS displayed a weak positive correlation (R = 0.173) with the indicator of Intra-Tumor Heterogeneity (ITH) at the pan-cancer level (Fig. 2E). However, this correlation demonstrated significant heterogeneity across different cancer types (Fig. 2F). For example, the DS showed a strong positive correlation with ITH-equ in COAD, Rectal Adenocarcinoma (READ), and Prostate Adenocarcinoma (PRAD), yet a negative correlation in Sarcoma (SARC). The positive correlation was particularly pronounced in COAD (R = 0.64, *P* < 0.001; Fig. 2G), suggesting that a high DS might coexist with higher tumor heterogeneity in specific cancer types.

Beyond the prognostic context, we systematically analyzed the association of the DS with genomic instability across the TCGA pan-cancer cohort. Pan-cancer regression analysis of the DS with mutation load unveiled significant cancer-type specificity (Fig. S2A). Remarkably, CRC and Ovarian Cancer (OV) exhibited the strongest, highly statistically significant positive correlation. Specifically, in the TCGA-COAD cohort, the DS showed a significant positive correlation with the Log2-transformed mutation load (R = 0.27, *P* < 0.001) (Fig. S2B). In stark contrast, the association analysis between the DS and the Copy Number Variation (CNV Score) presented an inverse trend (Fig. S2C). The majority of statistically significant cancer types (e.g., LUAD, BRCA, STAD, HNSC) were concentrated in the negative correlation quadrant. Moreover, refined analysis of somatic copy number variation (SCNV) at various scales (focal, chromosome arm, and whole-chromosome level) (Fig. S2D) universally supported the negative correlation trend between the DS and SCNV. Taken together, these findings suggest a complex, cancer-type-specific mechanism of regulation and co-existence between the dormancy state represented by the DS and these two major types of genomic instability (Mutation Load and CNV).

Then, we evaluated DS’s predictive performance for treatment response. Notably, the DS demonstrated robust predictive power for chemotherapy resistance across multiple independent clinical datasets, with AUC values consistently exceeding 0.6 and reaching up to 0.875 (Fig. 2H). Supporting this, cell line-based sensitivity analysis confirmed that high-DS populations exhibited significantly increased resistance to a broad spectrum of chemotherapeutic agents (Fig. S3A). This strongly substantiates the notion that tumor cells acquired multi-drug resistance to traditional broad-spectrum chemotherapies by activating the dormancy program [56]. In addition, the DS exhibited robust performance as a predictor for ICB therapy efficacy across four independent cohorts (Fig. S3B). In summary, the DS not only illuminates the mechanisms of tumor latency but also establishes itself as a powerful biomarker for predicting clinical resistance to both chemotherapy and immunotherapy.

### Construction and clinical validation of the COAD-specific dormancy score

**Fig. 3:**
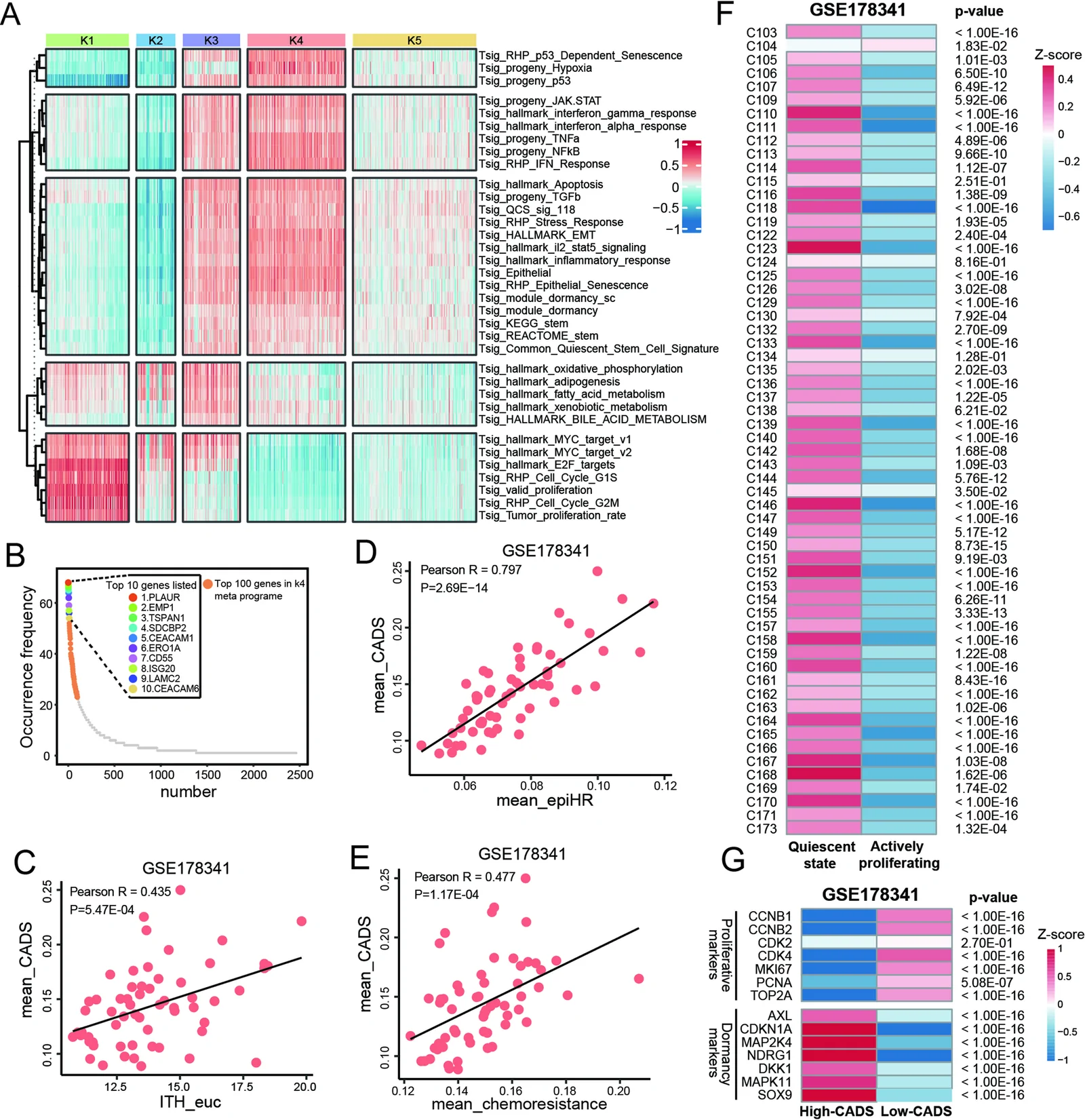
Integrative characterization of dormancy-associated meta-programs and validation of the CADS scoring system. **A** Heatmap showing the functional pathway activity patterns corresponding to distinct Non-negative Matrix Factorization (NMF) factors (K1-K5) in the colorectal cancer single-cell dataset GSE178341. Rows represent hierarchically clustered biological pathways (including cell cycle, inflammatory response, stress response, stemness, and metabolism), and columns represent cells/samples within each factor. Color intensity depicts the normalized pathway score (Z-score), with red indicating relatively high expression/activity and blue indicating low expression/activity. **B** Gene occurrence frequency statistics based on the NMF decomposition results, used to identify robust signature genes of the K4 factor (Dormancy-associated factor). Gray dots show the overall distribution of all genes, orange dots mark the top 100 genes with the highest occurrence frequency in the K4 meta-program, and the top 10 key genes are listed in the upper left corner. After removing genes that are duplicated with other programs, a total of 93 genes were selected as the CADS signature. **C**-**E** Correlation analysis between the mean COAD-specific Dormancy Score (mean_CADS) and biological features in GSE178341. CADS displays a significant positive correlation with intratumoral heterogeneity (ITH_euc) (**C**), the epithelial-specific high-risk gene set signature (mean_epiHR) (**D**), and the chemotherapy resistance score (mean_chemoresistance) (**E**). *P*-values indicate the statistical significance of the linear relationships (Pearson's correlation coefficient). **F** The tricycle method was used to calculate the cell cycle score, classifying cells into Quiescent or Actively proliferating states. The CADS distribution was compared between Quiescent and Actively proliferating cells in the GSE178341. The *P*-value confirms that Quiescent cells exhibit a significantly higher CADS than actively proliferating cells (Student's t-test). **G** Differential expression analysis of proliferation and dormancy markers based on CADS. Comparison of gene expression between the top 25% of malignant cells (High-CADS) and the bottom 25% of malignant cells (Low-CADS) based on CADS. The *P*-value indicates the statistical difference in gene expression (Student's t-test). NMF, Non-negative Matrix Factorization; CADS, COAD-specific Dormancy Score; ITH_euc, Intratumoral Heterogeneity (Euclidean distance metric); epiHR, epithelial-specific high-risk gene set signature

The focus on CRC was driven by its unique molecular and genomic profiles. Specifically, the DS exhibited a robust positive correlation with ITH-equ in CRC. Furthermore, pan-cancer regression analysis identified CRC as the cancer type with the strongest and most statistically significant positive correlation between the DS and mutation load. Together, these integrated findings suggest that the dormancy program may be a primary driver of genomic complexity and clinical heterogeneity in CRC, thereby warranting a detailed investigation into its underlying mechanisms in this specific malignancy. To systematically dissect the characteristics of the dormant state in CRC, we analyzed single-cell RNA sequencing (scRNA-seq) data from approximately 69,000 malignant cells (GSE178341). By applying the Non-negative Matrix Factorization (NMF) algorithm, we decoupled the malignant transcriptome into multiple gene expression programs and identified the K4 meta-programme as the foundational dormancy-associated signature (Fig. 3A). This meta-programme exhibited high positive correlations with single-cell dormancy modules, EMT, and inflammatory response phenotypes, representing a unique "dormancy-stemness-invasiveness" state. From the high-weight and high-frequency genes within K4, we constructed the core gene set for the CADS (Fig. 3B).

The identification of malignant cells used for this construction was strictly validated using the inferCNV algorithm, which confirmed extensive chromosomal amplification and deletion patterns in the GSE178341 patient samples (Fig. S4). Subsequent validation across internal and independent external cohorts (LEE, GSE166555) demonstrated that CADS is significantly and positively correlated with intratumoral heterogeneity (ITH), the epiHR recurrence signature [57], and chemotherapy tolerance (Fig. 3C-E, Fig. S4, Fig. S5A-B). These results imply that a high dormancy program is synchronized with increased clonal complexity and therapeutic resistance.

To validate the reliability of the CADS, we employed the tricycle method, which infers the cell cycle status of malignant cells based on the expression of cell cycle-related genes, classifying and quantifying cells into a quiescent state and an actively proliferating state [58]. We compared the statistical difference in CADS scores between quiescent cells and actively proliferating cells, as defined by the tricycle cycle score. The results showed that the CADS was significantly higher in quiescent cells compared to actively proliferating cells across all three datasets (Fig. 3F, Fig. S6A-B). This finding strongly confirms the biological reliability of the CADS as it can accurately distinguish between malignant cells in the two extreme states of proliferation and dormancy, independent of the cell cycle model. Simultaneously, we performed differential gene expression analysis on the top 25% and bottom 25% of malignant cells based on the CADS to identify specific molecular markers associated with dormancy and proliferation. In all three datasets (Fig. 3G, Fig. S6C-D), low-CADS cells significantly overexpressed key cell cycle and proliferation genes, including *CCNB1*, *CCNB2*, *CDK2*, *CDK4*, *MKI67*, *PCNA*, and *TOP2A*. Most differences were highly statistically significant. Conversely, high-CADS cells were significantly enriched for the expression of a series of known dormancy-related genes, including *AXL*, *CDKN1A*, *MAP2K4*, *NDRG1*, *DKK1*, *MAPK11*, and *SOX9*. The expression differences of these genes were highly significant in high-CADS cells.

### Biological validation of the CADS-high phenotype as a truly dormant and therapy-resistant population

To address whether the computationally derived CADS-high tumor population truly represents a bona fide "dormant" state rather than an active drug-tolerant or generalized stress-resistant state, we performed a series of rigorous in silico threshold evaluations and experimental validations using both mouse orthotopic models and patient-derived organoids (PDOs).

**Fig. 4:**
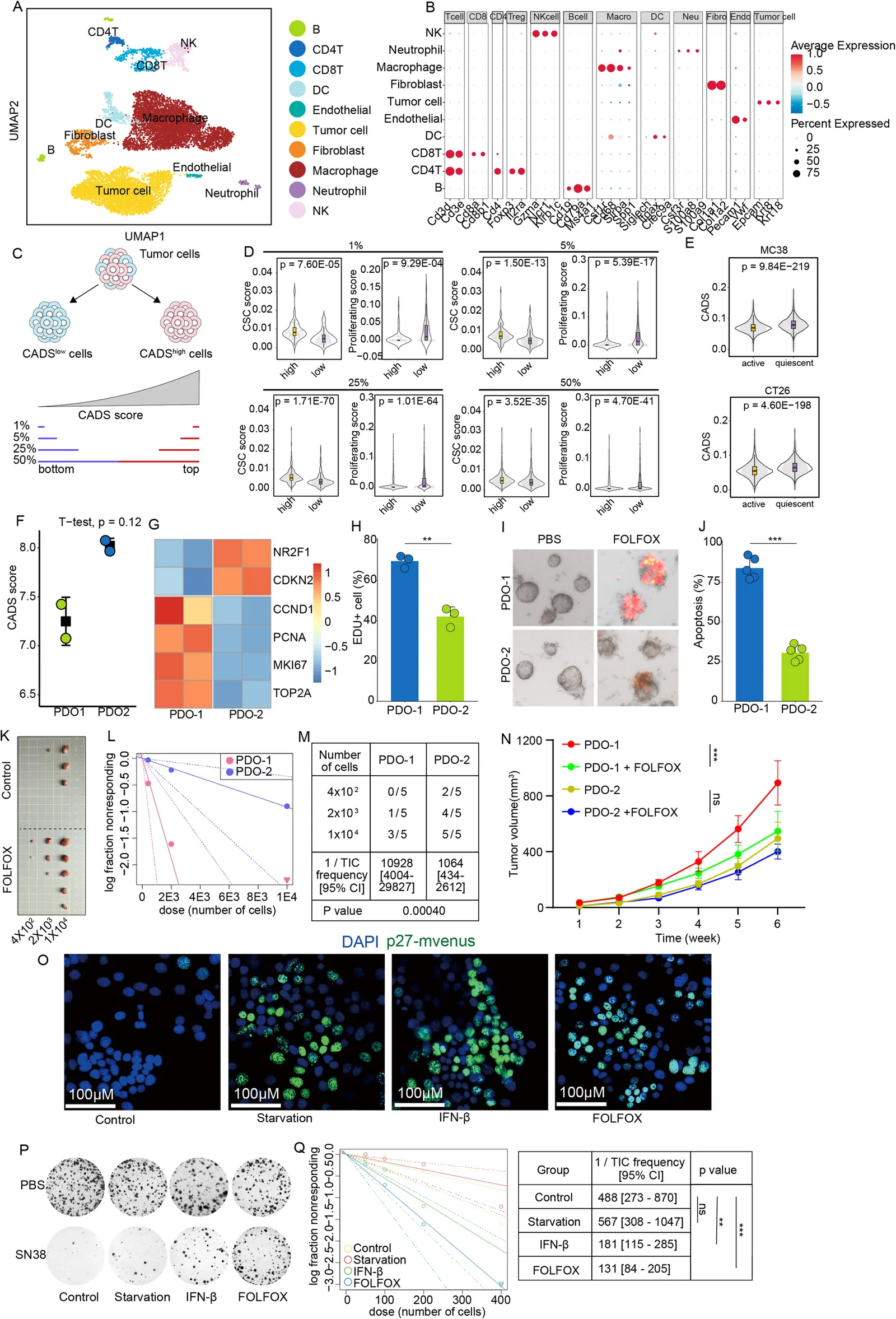
Functional Validation of CADS-Defined Dormancy and Distinct Microenvironmental Stress-Induced Dormant Subtypes in Colorectal Cancer. **A**–**B** Identification of major cell lineages on the orthotopic MC38 colon tumor. **A** UMAP visualization of single-cell transcriptomes, color-coded by cell type. **B** Dot plot illustrating the expression of canonical marker genes used for cluster annotation. **C**–**D** Evaluation of the CADS threshold robustness. **C** Schematic showing the grouping strategy based on different CADS score cutoffs (top vs. bottom 1%, 5%, 25%, and 50%). **D** Violin plots comparing CSC scores and Proliferating scores between CADS-high and CADS-low malignant cells across indicated thresholds in the MC38 orthotopic tumor model. *P*-values were determined by Student’s t-test. **E** Comparison of CADS between active and dormant malignant cell states in an independent CRC dataset (CNP0008774: MC38 and CT26 models). *P*-values indicate statistical significance via Student’s t-test. **F**–**J** In vitro validation of the dormant phenotype using Patient-Derived Organoids (PDOs). **F** CADS distribution in two representative CRC-PDO models (PDO-1 and PDO-2). **G** Heatmap of transcriptomic data showing the expression of dormancy markers (NR2F1, CDKN2A) and proliferation markers (CCND1, PCNA, MKI67, TOP2A) in PDOs. **H**–**J** Functional assays of PDOs. **H** Statistics of EdU^+^ proliferating cells. **I** Representative bright-field and fluorescence images of PDOs treated with PBS or FOLFOX. **J** Quantification of apoptosis rates following FOLFOX treatment. **K**–**N** In vivo validation of tumor-initiating capacity and chemoresistance. **K** Representative images of tumors harvested from nude mice injected with varying cell doses (4 × 10^2^, 2 × 10^3^, 1 × 10.^4^) of PDO-1 or PDO-2, treated with or without FOLFOX. **L**–**M** Extreme Limiting Dilution Analysis (ELDA) calculating the Tumor-Initiating Cell (TIC) frequency. **N** Tumor growth kinetics over 6 weeks. **O** Representative immunofluorescence images of MC38 cells expressing the p27-GFP (green). Different methods were used to induce dormancy including starvation (serum deprivation), IFN-β treatment, or in vivo FOLFOX treatment. DAPI (blue) was used for nuclear counterstaining. Scale bar = 100 μM. **P** Colony formation assays evaluating the sensitivity to SN38 (active metabolite of irinotecan) in MC38 cells pre-conditioned with indicated dormancy-inducing stressors. Note the significantly higher survival in IFN-β and FOLFOX-primed groups compared to the starvation group. **Q** Extreme Limiting Dilution Analysis (ELDA) assessing the tumor-initiating cell (TIC) frequency of MC38 cells from different groups. The log-fraction of non-responding wells is plotted against the number of cells seeded. The table (right) provides the calculated TIC frequencies with 95% confidence intervals (CI). Data are presented as mean ± SD. ***P < 0.001, *P* < 0.01; ns, non-significant (Two-way ANOVA for tumor growth; ELDA for TIC frequency)

We conducted single-cell RNA sequencing on an orthotopic MC38 colon tumor model, identifying major cell lineages including malignant cells, T cells, B cells, myeloid cells, and fibroblasts via canonical markers (Fig. 4A–B). To rationalize the mathematical threshold for defining "dormancy" within the tumor mass, we implemented a threshold robustness analysis by dividing malignant cells into CADS-high and CADS-low groups based on varying top/bottom quantiles (1%, 5%, 25%, and 50%) (Fig. 4C). Intriguingly, stratified profiling revealed that even when the cutoff was broadened from the stringent 1% to 25%, the CADS-high malignant cells consistently maintained significantly elevated cancer stem cell (CSC) scores and markedly suppressed proliferation scores (Fig. 4D). However, at the 50% threshold, these dormancy-associated traits became significantly attenuated, demonstrating that a 25% threshold serves as an optimal and balanced cutoff that captures robust quiescent properties while ensuring adequate cell numbers for statistical reliability. This biological relevance was further corroborated in an independent CRC single-cell dataset (CNP0008774) [49], where cells designated as dormant exhibited significantly higher CADS compared to their active counterparts (Fig. 4E).

To functionally substantiate these findings in human preclinical models, we selected two representative PDOs displaying distinct CADS distributions: PDO-1 (low-CADS) and PDO-2 (high-CADS) (Fig. 4F). Transcriptomic benchmarking via bulk RNA-seq revealed a stark divergence in molecular phenotypes; the low-CADS PDO-1 highly expressed classical proliferation markers (CCND1, PCNA, MKI67, and TOP2A), whereas the high-CADS PDO-2 was markedly enriched with established dormancy orchestrators, including CDKN2A and NR2F1 (Fig. 4G). In alignment with the transcriptomic profiles, EdU incorporation assays demonstrated a significantly lower percentage of proliferating cells in high-CADS PDOs (Fig. 4H). Crucially, functional modeling under chemotherapeutic stress showed that high-CADS PDOs possessed heightened resistance to FOLFOX treatment, characterized by a prominent reduction in therapy-induced apoptosis rates compared to low-CADS PDOs (Fig. 4I–J).Finally, we evaluated the in vivo tumor-initiating capacity (TIC) and growth kinetics of these populations using extreme limiting dilution assays (ELDA) in nude mice injected with graded cell doses (4 × 10^2^, 2 × 10^3^, 1 × 10^4^ cells) and subjected to vehicle or FOLFOX challenges (Fig. 4K). Under baseline conditions, high-CADS PDOs exhibited a significantly higher TIC frequency alongside intrinsically slower, indolent tumor growth kinetics over a 6-week period (Fig. 4L–N). Under therapeutic pressure, while FOLFOX dramatically suppressed the growth of low-CADS tumors, high-CADS-derived tumors displayed profound chemoresistance, continuing their slow but unperturbed progression (Fig. 4N). Taken together, these comprehensive in vitro and in vivo functional matrices definitively demonstrate that a high CADS score successfully identifies a specialized, slow-cycling, stem-like, and therapy-resistant dormant tumor cell population in colorectal cancer.

To further investigate whether this observed trade-off between proliferation and malignancy is a universal feature of all dormant states, we systematically compared MC38 cells under three distinct microenvironmental stressors: starvation (serum deprivation), IFN-β treatment, and in vivo FOLFOX challenge (Fig. 4O). While all stimuli successfully triggered cell cycle arrest, as evidenced by the accumulation of the p27-mVenus dormancy reporter (Fig. 4O), their functional impacts on tumor fitness were markedly divergent. Notably, colony formation and ELDA assays revealed that dormancy induced by therapeutic or immune stress (IFN-β and FOLFOX) promoted a significant enrichment in tumor-initiating cell (TIC) frequency and chemoresistance (Fig. 4P-Q). In contrast, starvation-induced dormancy resulted in a relatively inert phenotype with reduced survival capacity under subsequent drug challenge. These data suggest that the "Go-or-Grow" trade-off is not an intrinsic property of all quiescent cells but rather a selective adaptive strategy specifically employed by cancer cells to survive immune or therapeutic pressure.

### CADS defines the immunological landscape of MMRd/MSI-H colorectal cancer and predicts immunotherapy response

After establishing that the CADS effectively characterizes the dormancy traits of malignant cells and its role in remodeling the immune microenvironment, we further investigated the distribution of this dormancy program across clinical pathological subtypes. Given that mismatch repair-deficient (MMRd) or microsatellite instability-high (MSI-H) colorectal cancers exhibit distinct immunogenicity and clinical outcomes, we sought to elucidate whether the dormant cell state is a pivotal factor driving the unique immune features of these subtypes.

**Fig. 5:**
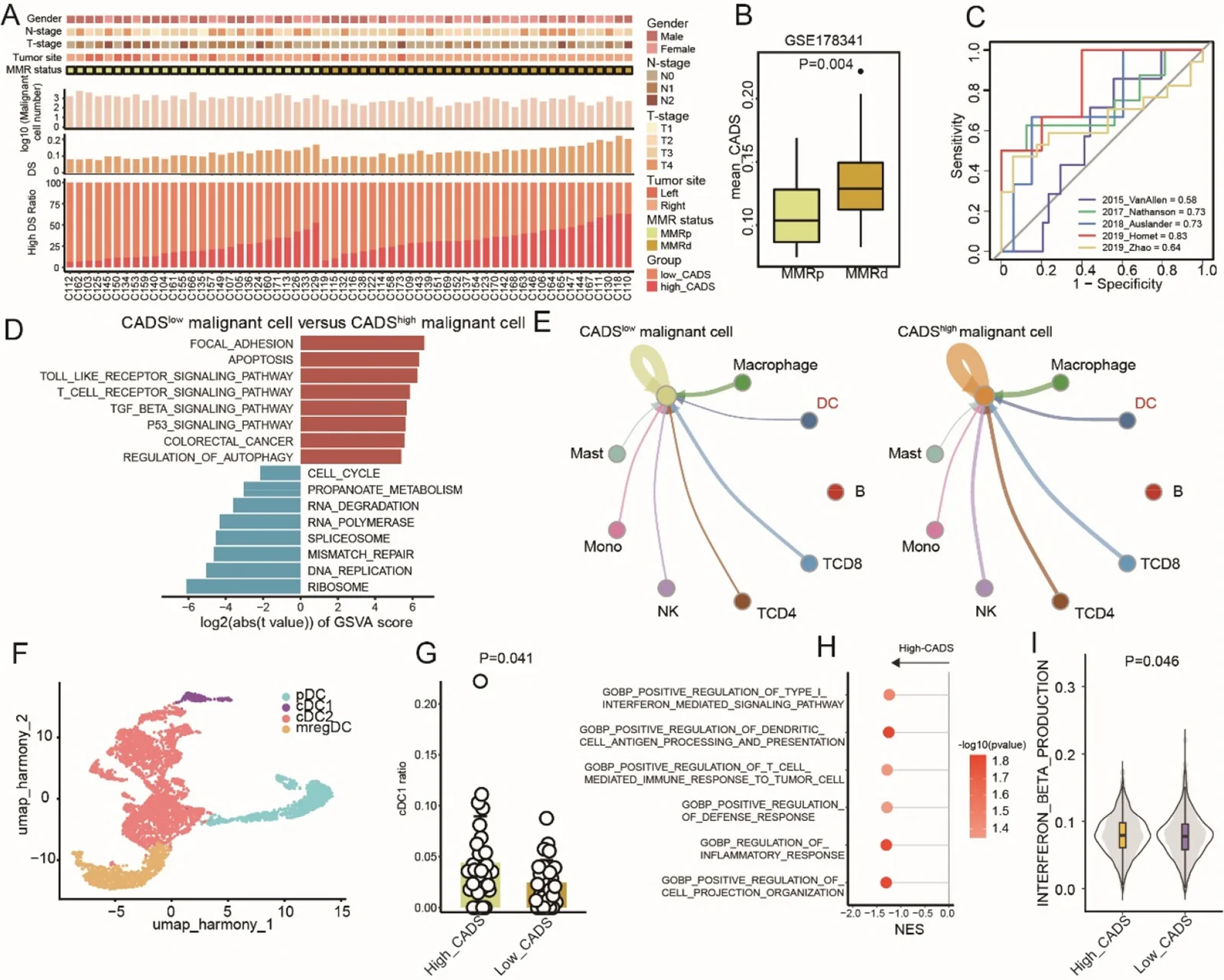
Multi-omic characterization of the interplay between dormant malignant cells and the immune landscape across clinical cohorts. **A** Overview of clinicopathological and molecular features for the 60 patients in the GSE178341 dataset, showcasing gender, TNM stage, tumor location, MMR status, tumor site, and CADS distribution per sample. **B** Comparison of mean CADS between mismatch repair-proficient (MMRp) and -deficient (MMRd) tumors. Results show that the MMRd group has a significantly higher dormancy signature score (*P* = 0.004, Student's t-test). **C** Receiver operating characteristic (ROC) curves evaluating the efficacy of CADS in predicting immunotherapy response across multiple independent clinical cohorts. Curves depict sensitivity versus specificity in the VanAllen, Nathanson, Homet studies and etc. **D** Gene Set Variation Analysis (GSVA) comparing pathway activity differences between CADS-low and CADS-high malignant cells. **E** Cell–cell communication analysis depicting the interaction network patterns between CADS-low and CADS-high malignant cells and immune cells (including monocytes, macrophages, dendritic cells, T cells, and NK cells). **F** Clustering analysis of all dendritic cells (DC) based on single-cell transcriptomic data identified four main cell subpopulations: conventional DC type 1 (cDC1), conventional DC type 2 (cDC2), plasmacytoid DC (pDC), and regulatory DC (mregDC) characterized by regulatory features. **G** Comparison of the relative percentage of cDC1 within tumors between patients in the CADS-low group and the CADS-high group. **H** Gene Set Enrichment Analysis (GSEA) of transcriptomic data comparing the CADS-high group versus the CADS-low group. Results show significant enrichment for pathways including type I interferon response, antigen processing and presentation, and T cell-mediated anti-tumor immunity (Normalized Enrichment Score (NES) and *P* value are used to determine statistical significance). **I** Violin plot comparing the interferon-beta (IFN-β) production signature levels between the CADS-high and CADS-low groups. The CADS-high group exhibits significantly higher IFN-beta levels (*P* = 0.046, Student's t-test). NCADS, COAD-specific Dormancy Score; MMR, Mismatch Repair; GSVA, Gene Set Variation Analysis; DC, Dendritic Cell; cDC1/cDC2, Conventional Dendritic Cell Type 1/2; pDC, Plasmacytoid Dendritic Cell; mregDC, Regulatory Dendritic Cell; GSEA, Gene Set Enrichment Analysis; ROC, Receiver Operating Characteristic; NES, Normalized Enrichment Score; FDR, False Discovery Rate

Our analysis of clinical samples revealed significant inter-patient heterogeneity in CADS. Notably, CADS was significantly higher in MMRd tumors compared to mismatch repair-proficient (MMRp) tumors (*P* = 0.004; Fig. 5A-B). To validate the universality of this association, we conducted parallel analyses across multiple independent external cohorts. In the TCGA-COAD cohort, MSI-H tumors exhibited significantly elevated CADS scores compared to MSI-L/MSS tumors (*P* = 0.046; Fig. S7A). This trend was further confirmed in the larger GSE39582 cohort, where MMRd tumors again demonstrated significantly higher CADS scores than MMRp tumors (*P* = 0.021; Fig. S7B). Our findings establish a strong link between dormancy and the MMRd/MSI-H phenotype, suggesting that the dormant cell state may represent a previously unrecognized factor contributing to the high immunogenicity and distinct clinical outcomes observed in this patient subgroup.

Crucially, CADS demonstrated robust predictive performance for immunotherapy response in several clinical cohorts, including the VanAllen and Homet datasets, underscoring the critical role of dormant cell states in shaping the immune microenvironment (Fig. 5C). To clarify the specific mechanisms by which CADS influences immune responses, we compared the molecular profiles of high-CADS and low-CADS malignant cells using gene set variation analysis. Low-CADS (proliferative) cells were markedly enriched in pathways associated with the cell cycle, DNA replication, and basal metabolism, such as CELL_CYCLE, DNA_REPLICATION, and RIBOSOME. In contrast, high-CADS cells displayed activation of pathways supporting cell survival, invasion, and immune regulation, including FOCAL_ADHESION, TGF_BETA_SIGNALING, P53_SIGNALING, TOLL_LIKE_RECEPTOR_SIGNALING, and REGULATION_OF_AUTOPHAGY (Fig. 5D). Furthermore, cell–cell communication analysis revealed that high-CADS malignant cells established a more complex and intensive interaction network with various immune populations, including macrophages, DCs, and T cells (Fig. 5E).

### Single-cell dissection of CADS and tumor microenvironment interaction

To systematically resolve the mechanisms by which dormant malignant cells reshape the immune microenvironment, we conducted high-resolution single-cell transcriptomic profiling of tumor-infiltrating DCs and CD8^+^ T cells. We specifically assessed how elevated CADS scores influence the functional states of DCs, the primary antigen-presenting cells within the tumor milieu. DCs from integrated patient samples (C103-C173) were visualized via UMAP (Fig. 5F).

We identified and annotated four distinct DC subsets based on canonical markers: conventional type I DCs (cDC1), conventional type II DCs (cDC2), plasmacytoid DCs (pDC), and mature regulatory DCs (mregDC) characterized by high expression of regulatory genes (Fig. S8A). Quantitative assessment demonstrated that the proportion of cDC1 was significantly elevated in the high-CADS group compared to the low-CADS group (*P* = 0.041; Fig. 5G), while the proportions of cDC2, pDC, and mregDC did not show significant differences between the two groups (Fig. S8B-D).

Further GSEA enrichment analysis revealed that the high-CADS group was significantly enriched for pathways involved in the positive regulation of type I interferon-mediated signaling, DC antigen processing and presentation, and T cell-mediated anti-tumor immune responses (Fig. 5H). Consistently, the elevation of IFN-β production signatures in the high-CADS group (*P* = 0.046, Fig. 5I) underscores the pivotal role of dormant tumor cells in modulating interferon signaling and driving subsequent immune activation. Dormant malignant cells orchestrate the immune microenvironment by secreting IFN-β, which triggers the activation of type I interferon signaling and the specific recruitment and activation of the cDC1 subset, thereby enhancing tumor antigen presentation and T cell-mediated anti-tumor immune responses. While cellular dormancy is traditionally associated with immune evasion due to low proliferative activity, our findings demonstrate that in colorectal cancer, the dormant subpopulation (CADS-high) acts as a "coordinator" of the immune milieu. By maintaining a functional IFN-β-cDC1 axis, these cells sustain the microenvironment in a "primed" state, ready to mount a robust immune response.

To support this point, we performed GSVA on normalized spatial transcriptomics data. We focused on three biologically relevant gene signatures: cDC1, associated with dendritic cell infiltration; CADS, linked to tumor cell dormancy; and IFN-β signaling. GSVA scores revealed distinct spatial patterns of signature enrichment across multiple tissue sections (Fig. S8E). we observed pronounced co-enrichment of cDC1 and IFN-β signatures, alongside elevated CADS, indicating a spatially coordinated interplay between immune recruitment and dormancy programs. These findings highlight the spatial heterogeneity of immune and dormancy signals within tumors and suggest that localized microenvironmental niches may differentially regulate tumor-immune interactions.

To investigate the relationship between CADS and the functional polarization or exhaustion status of effector T cells, we utilized UMAP dimensional reduction to categorize CD8^+^ T cells into seven distinct functional states: effector (CD8_eff), exhausted (CD8_ex), naive (CD8_naive), central memory (CD8_Tcm), effector memory (CD8_Tem), resident memory (CD8_Trm), and proliferating (CD8T_proliferating) (Fig. S8F-G).

Proportional analysis revealed that patients in the low-CADS group possessed a significantly higher percentage of central memory T cells (Tcm) compared to the high-CADS group (*P* = 0.002) (Fig. S8H-I). However, GSEA indicated that T cells within the high-CADS environment were significantly enriched for pathways including REGULATION_OF_T_CELL_ACTIVATION, REGULATION_OF_RESPONSE_TO_CYTOKINE_STIMULUS, and notably, RESPONSE_TO_INTERFERON_BETA (Fig. S8J).

Functional scoring further demonstrated that T cells in the high-CADS group exhibited substantially higher cytotoxicity scores (*P* = 2.2E-16) and elevated naive/memory-related scores (*P* = 2.9E-4) compared to their low-CADS counterparts (Fig. S8K-L). These findings suggest that while high dormancy features correlate with a reduction in traditional central memory proportions, they paradoxically foster a T cell population with enhanced cytotoxic potential and heightened responsiveness to interferon-beta.

### Anti-PD1 enhances immune environment and induces tumor dormancy

**Fig. 6:**
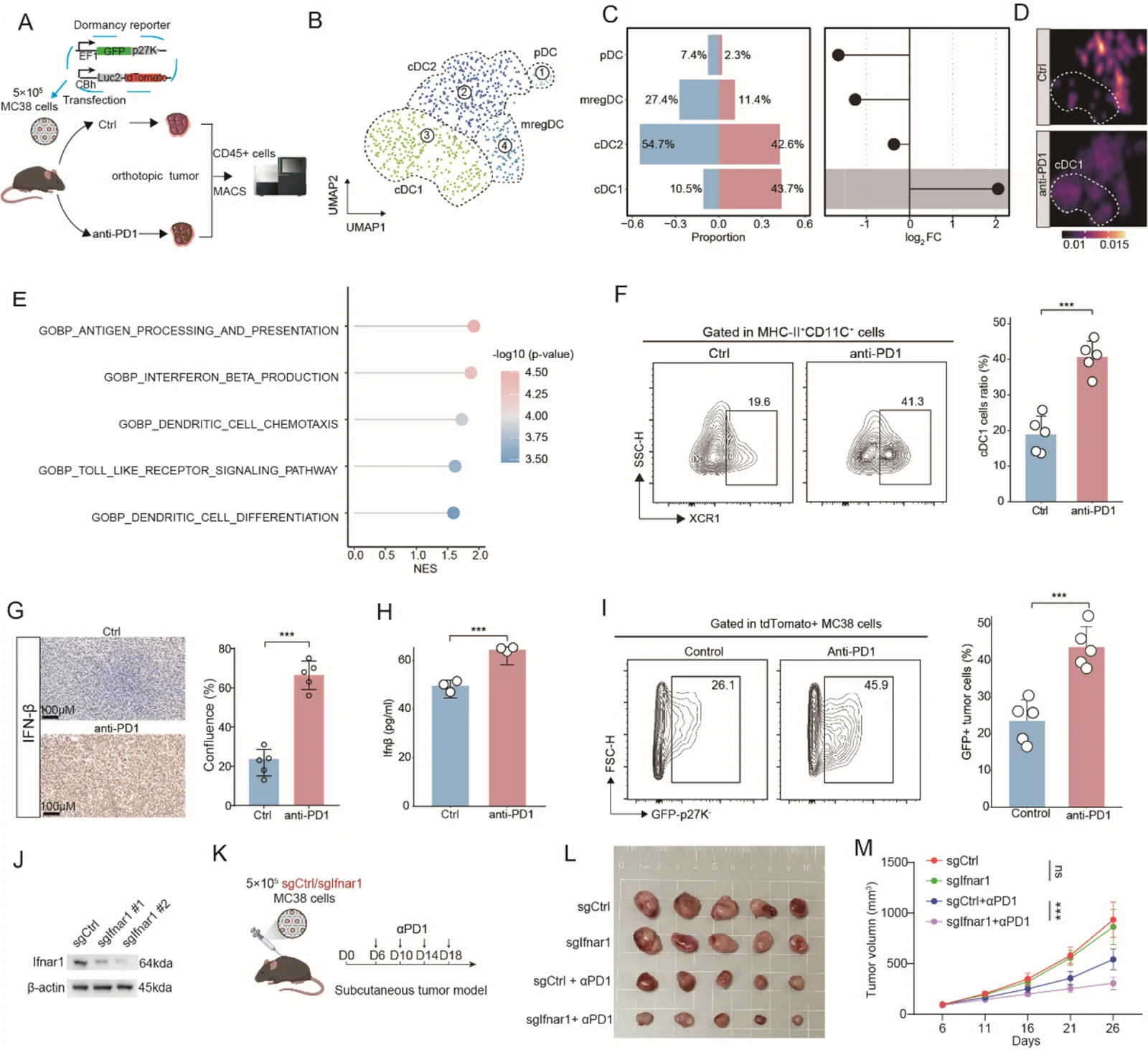
Anti-PD-1 Elicits IFN-β Dependent Anti-tumor Immunity and DC Reprogramming. **A** Mouse experimental scheme. MC38 cells transfected with a dormancy reporter were injected to form tumors, followed by anti-PD1 treatment. CD45^+^ cells were then isolated via magnetic bead sorting and analyzed by single-cell sequencing. **B** UMAP subset clustering analysis of DC cell populations before and after anti-PD1 treatment, identifying four subsets: pDC, cDC2, cDC1, and mregDC. **C** Comparison of the relative proportions and log2FC of DC subsets in the Ctrl and anti-PD1 groups. Anti-PD1 treatment resulted in a significant increase in the cDC1 proportion (from 10.5% to 43.7%). **D** Distribution of the cDC1 subset in the UMAP space before and after anti-PD1 treatment. **E** GOBP pathway enrichment analysis of cDC1 cells enriched following anti-PD1 treatment. The *P*-value determines the statistical significance of the enrichment (Gene Set Enrichment Analysis (GSEA)). **F** Flow cytometry detection of the proportion of XCR1 + cDC1 cells within the MHC-II + CD11c + population. Anti-PD1 treatment significantly increased the cDC1 proportion from 19.6% to 41.3% (*P* < 0.001, Student's t-test was used to compare the means). **G** Mouse immunohistochemistry (IHC) showing increased IFN-β expression after anti-PD1 treatment. Scale bar = 100 μm. **H** ELISA showing the change in IFN-β protein expression in mouse tumors before and after anti-PD1 treatment. The *P*-value indicates a statistically significant difference (Student's t-test). **I** Flow cytometry detection of the change in the GFP-p27K positive cell proportion before and after anti-PD1 treatment. The *P*-value indicates a statistically significant difference (Student's t-test). **J** sgRNA-mediated Ifnar1 protein knockdown validation. **K** Experimental scheme. MC38 cells with Ifnar1 knockout were injected to form subcutaneous tumors, followed by anti-PD1 blockade treatment. **L**-**M** Tumor volume curve in an immunocompetent mouse colorectal cancer model. The sgIfnar1 group combined with anti-PD1 treatment showed a significantly larger tumor volume than the sgCtrl + anti-PD1 group (*P* < 0.001, Two-way ANOVA with repeated measures). anti-PD-1, anti-programmed cell death protein 1; IFN-β, interferon-beta; DC, dendritic cell; cDC1, conventional dendritic cell type 1; pDC, plasmacytoid dendritic cell; cDC2, conventional dendritic cell type 2; mregDC, regulatory dendritic cell; GFP-p27K, green fluorescent protein-tagged p27Kip1 dormancy reporter; Ifnar1, interferon-alpha/beta receptor subunit 1; UMAP, uniform manifold approximation and projection; GOBP, Gene Ontology Biological Process; GSEA, gene set enrichment analysis; IHC, immunohistochemistry; ELISA, enzyme-linked immunosorbent assay; sgRNA, single-guide RNA; ANOVA, analysis of variance

To elucidate the effect of anti-PD1 ICB on the colon cancer TME, we conducted in vivo studies using the syngeneic MC38 mouse tumor model. Following anti-PD1 treatment, tumor-infiltrating CD45^+^ cells were isolated for single-cell RNA sequencing (scRNA-seq) analysis (Fig. 6A). Analysis of major immune cell populations revealed a significant shift in TME composition after anti-PD1 treatment (Fig. S9A-B). Specifically, the proportion of cytotoxic T cells (CD8^+^T) significantly increased from 10.4% in the control group to 24.6%, while the proportion of DCs also rose sharply (from 1.4% to 7.2%) (Fig. S9C-D).

Functionally, anti-PD1 treatment significantly improved the functional state of CD8^+^T cells, evidenced by a dramatic migration and enrichment of the T cell population towards the lower-right quadrant (low exhaustion/high fitness). This indicates that the treatment successfully activated T cells, moving them out of the exhausted state (decreased Exhaustion Score) and gaining high function/fitness (increased Fitness Score) (Fig. S9E).

Given the crucial role of Antigen Presenting Cells (APCs) in T cell priming, we analyzed DCs. UMAP visualization distinguished four different DC/myeloid subsets: cDC1, cDC2, pDC, and a regulatory macrophage/DC population (mregDC) (Fig. 6B). Quantitative analysis of DC subsets (Fig. 6C-D) revealed that anti-PD1 treatment has a highly selective and profound impact. The proportion of cDC1 cells increased markedly from 10.5% in the control group to 43.7% following anti-PD-1 treatment, indicating a pronounced expansion of this subset within the dendritic-cell lineage.

In light of the significant cDC1 enrichment, we utilized GSEA analysis to investigate the enriched functional pathways in the DC population after anti-PD1 treatment. We observed that Antigen_Processing_and_Presentation showed the highest enrichment in the anti-PD1-enriched cDC1 cells, emphasizing the enhancement of APC function. Interferon_Beta_Production was highly statistically significant, suggesting the activation of the Type I Interferon (IFN-I) response. Pathways related to DC differentiation and migration, such as Dendritic_Cell_Chemotaxis and Dendritic_Cell_Differentiation, were also positively enriched. This indicates that cDC1 plays a core role in enhancing tumor antigen presentation (Fig. 6E).

We subsequently validated the function of cDC1 enrichment and IFN-I signaling upregulation. We confirmed cDC1 enrichment using flow cytometry, which showed that the proportion of MHC-II^+^CD11c^+^XCR1^+^ cells (the hallmark population of cDC1) significantly increased in the anti-PD1 group compared with the control group (*P* < 0.001) (Fig. 6F). Immunohistochemistry staining showed a significant increase in IFN-β expression in tumor tissues after anti-PD1 treatment. ELISA also confirmed the increase in IFN-β protein concentration, proving the activation of the IFN-I pathway (Fig. 6G-H, P < 0.001).

To assess the impact of anti-PD1 treatment on tumor cell fate, particularly the cellular dormant state, we used MC38 cells expressing both tdTomato and the dormancy reporter GFP-P27K^−^ [59–61], in which GFP-positive cells indicate tumor cells in a dormant state. Anti-PD1 treatment significantly increased the proportion of GFP^+^ (dormant) tumor cells, rising from 26.1% in the control group to 45.9% (*P* < 0.001) (Fig. 6I). This suggests that under the pressure of a highly effective anti-tumor immune response (such as anti-PD1 treatment), a subset of tumor cells may not be eliminated but enter an adaptive dormant state. This dormancy is likely a survival strategy adopted by tumor cells to evade immune-mediated killing and extrinsic stress imposed by the TME. Therefore, while anti-PD1 therapy enhances immune killing, it may simultaneously enrich for residual tumor cells in the dormant state, which could be relevant to the commonly observed mechanisms of clinical resistance and recurrence.

To explore the functional necessity of the IFN-I pathway, we engineered a CRISPR/Cas9 knockout of the Type I Interferon α/βReceptor, Ifnar1, in the MC38 cell line (Fig. 6J). Figure 6K-L illustrates the experimental setup and endpoint tumor volume. Wild-type (sgCtrl) or sgIfnar1 tumors received anti-PD1 treatment. To determine the effect of IFN-I signaling for tumor cells in affecting anti-PD1 efficacy, we compared the growth of wild-type and IFNAR1 knockout tumors with or without anti-PD1 treatment (Fig. 6M). sgCtrl and sgIfnar1 tumors showed very similar growth curves without treatment, with no significant statistical difference. This indicates that the absence of Ifnar1 itself does not affect the tumor's basal growth rate. However, the Ifnar1 loss significantly enhanced the therapeutic effect of PD1 blockade. These observations suggest that IFN-I signaling in tumor cells may constitute a major inhibitory factor that limits the efficacy of PD-1 blockade.

### IFN-β induces dormancy, leading to differential drug sensitivity

Given that the efficacy of anti-PD1 critically depends on Type I Interferon (IFN-I) signaling, we investigated the direct effects of IFN-β on CRC cell lines (HCT116 and SW620).

**Fig. 7:**
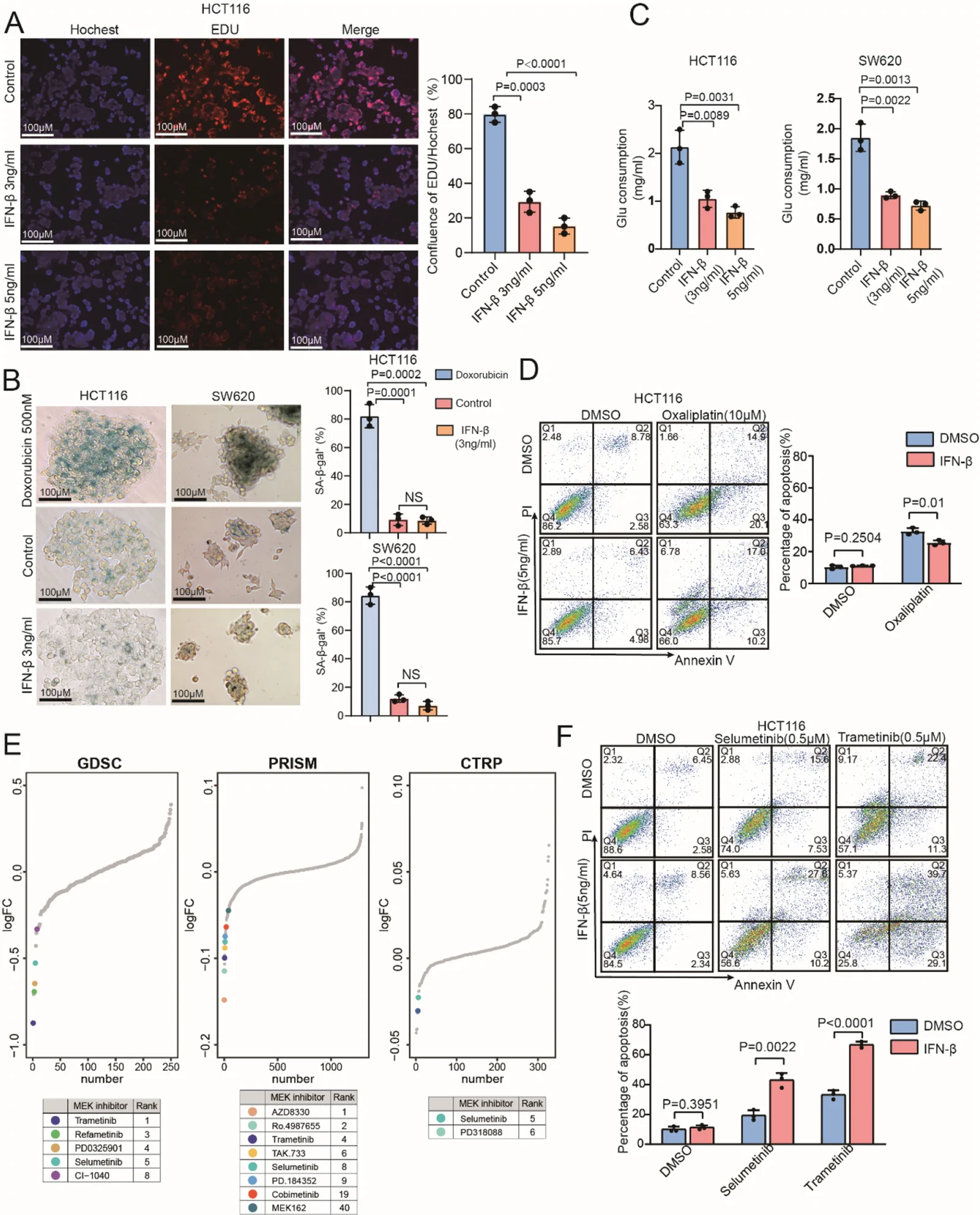
IFN-beta Induces Tumor Cell Quiescence and Increases Sensitivity to MEK Inhibitors. **A** EdU incorporation assay evaluating the proliferative capacity of HCT116 cells treated with indicated concentrations of IFN-β (3 ng/ml and 5 ng/ml). Representative immunofluorescence images of EdU and Hoechst staining (left) and quantification of EdU/Hoechst positive cells (right) are shown. Scale bar = 100 μm. **B** Microscopic Imaging (Left): Representative bright-field images of HCT116 and SW620 cells. Treatment conditions include Control, IFN-β (3 ng/ml), and Doxorubicin (500 nM) serving as a positive control for senescence. Scale bars = 100 μm. Quantitative Analysis (Right): Bar graphs showing the percentage of SA-β-gal-positive cells. Data are presented as mean ± SD from three independent experiments. Scale bar = 100 μm. **C** Glucose consumption assay showing the change in glucose uptake in HCT116 and SW620 cells after IFN-β treatment. The *P*-value indicates a statistically significant difference (Student's t-test). **D** Flow cytometric analysis of apoptosis using Annexin V and PI staining in HCT116 cells treated with IFN-β (5 ng/ml) alone or in combination with Oxaliplatin (10 μM). The bar graph (right) represents the percentage of apoptotic cells. **E** Pharmacological sensitivity ranking of dormancy-like cancer cells across GDSC, PRISM, and CTRP databases. MEK inhibitors consistently ranked among the most effective compounds (lowest logFC) in cells exhibiting dormant features. **F** Synergistic effect of IFN-β and MEK inhibitors (Selumetinib and Trametinib) on cell apoptosis. HCT116 cells were analyzed by Annexin V/PI double staining after combination treatments, with quantitative data shown in the lower panel (Student's t-test). IFN-β: Interferon-beta; EDU: 5-ethynyl-2'-deoxyuridine; SA-β-gal: Senescence-Associated β-galactosidase; NS: Not Significant; GDSC: Genomics of Drug Sensitivity in Cancer; PRISM: Profiling Relative Inhibition Subsets in Multiple myeloma; CTRP: Cancer Therapeutics Response Portal; MEK: Mitogen-activated protein kinase kinase; PI: Propidium Iodide

Previous studies have shown that IFN-β can induce tumor dormancy, thereby conferring enhanced drug resistance and tumor stem cell-like properties [62]. Through EDU/Hoechst double staining, we found a sharp suppression of EDU incorporation (i.e., DNA synthesis and proliferation) in HCT116 cells after treatment with different concentrations of IFN-β. Quantitative analysis confirmed that IFN-β significantly reduced the percentage of proliferating HCT116 cells compared to the control group (*P* < 0.001) (Fig. 7A). However, SA-β-gal staining results showed that IFN-β treatment did not significantly induce senescence in CRC cells (Fig. 7B), indicating that its anti-proliferative effect is not mediated by the senescence pathway.

To explore the mechanism by which IFN-β inhibits cell growth, we assessed its impact on cell metabolism. Figure 7C showed that IFN-β treatment significantly reduced glucose consumption in both HCT116 and SW620 cells. These results suggest that IFN-β can act directly on tumor cells, exerting its intrinsic anti-tumor function by inhibiting key glucose metabolic activity in addition to cell proliferation.

In the clinical management of CRC, oxaliplatin serves as a cornerstone of first-line chemotherapy regimens, such as FOLFOX, and is widely recognized for its capacity to induce immunogenic cell death, thereby priming anti-tumor immune responses [63]. We therefore reasoned that the addition of IFN-beta might synergize with oxaliplatin to enhance cytotoxicity. However, contrary to this expectation, flow cytometric analysis revealed that the combination of 5 ng/ml IFN-β and 10 μM oxaliplatin led to a significant reduction in the proportion of apoptotic cells compared to oxaliplatin monotherapy in both HCT116 and SW620 cell lines (Fig. 7D and Fig. S10A). These counter-intuitive findings suggest that, under current experimental conditions, IFN-β may exert a paradoxical protective effect on CRC cells-potentially by inducing a state of dormancy-which subsequently diminishes their sensitivity to oxaliplatin-mediated chemotherapy. Thus, to explore the vulnerability of IFN-β induced dormant cells, we conducted drug sensitivity screening based on GDSC, CTRP, and PRISM databases, and the results showed bioinformatic analysis based on the GDSC, CTRP, and PRISM databases for drug sensitivity screening suggested that the dormancy pattern significantly altered the response profile to MEK inhibitors (Fig. 7E): exhibiting increased sensitivity to multiple MEK inhibitors (such as Trametinib, Selumetinib, etc.). We assessed the apoptotic effects of IFN-β in combination with MEK inhibitors (Selumetinib, Trametinib) using Annexin V/PI staining. The combined use of IFN-β with MEK inhibitors Selumetinib and Trametinib induced significantly higher apoptosis rates than monotherapy, demonstrating a powerful synergistic anti-tumor activity between IFN-β and MEK inhibitors (Fig. 7F, Fig. S10B). In contrast, when IFN-β was combined with the DNA-damaging chemotherapy drug Oxaliplatin, the apoptosis rate was significantly lower than expected or showed an antagonistic trend. Although the apoptosis rate in the combination group was still higher than the control group, IFN-β attenuated the intrinsic apoptotic effect of Oxaliplatin itself. This differential sensitivity to various drugs strongly suggests that the cell fate transition induced by IFN-β plays a core role: Oxaliplatin's primary mechanism of action is the formation of DNA adducts, exerting maximal cytotoxicity during cell division. When IFN-β pushes tumor cells into a non-proliferative dormant state, these cells acquire inherent resistance to Oxaliplatin killing. Therefore, IFN-β actually antagonizes the cytotoxicity of Oxaliplatin by promoting cell dormancy. Conversely, while dormant cells escape Oxaliplatin killing, they still rely on specific survival signaling pathways to maintain cell viability and prevent apoptosis. Since the MAPK/MEK pathway plays a crucial role in maintaining the survival of non-proliferative cells, MEK inhibitors can efficiently target and eliminate this subset of residual cells induced into dormancy by IFN-β, thereby producing a powerful synergistic killing effect.

### Combined blockade synergistically overcomes adaptive dormancy

Based on the preceding findings, we hypothesized that effective anti-PD1 immune blockade, while inhibiting tumor cell proliferation via IFN-β signaling, might simultaneously drive tumor cells into an adaptive dormant state through this IFN-β-induced non-proliferative state, becoming a source of resistance and recurrence. To validate this hypothesis and develop a strategy to break this mechanism, we designed a combination therapy regimen using the MEK inhibitor Trametinib and anti-PD1 therapy.

**Fig. 8:**
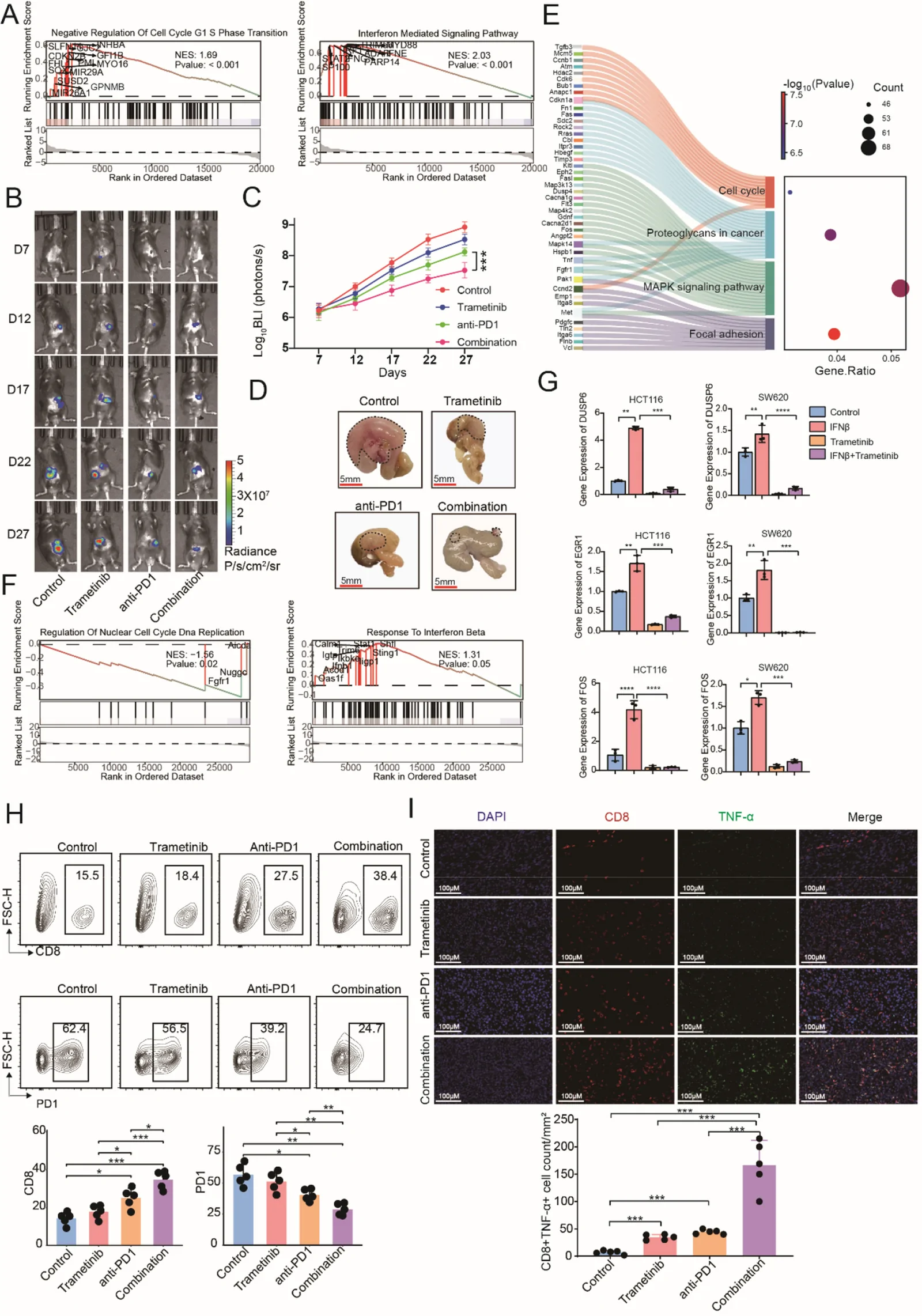
Synergistic Anti-tumor Efficacy of IFN-beta-Induced Combination Therapy in vivo. **A** Gene Set Enrichment Analysis (GSEA) showing enrichment of the "Negative Regulation of Cell Cycle G1 S Phase Transition" pathway (NES = 1.69, *P* < 0.001, GSEA was performed) and significant enrichment of the "Interferon Mediated Signaling Pathway" (NES = 2.03, *P* < 0.001, GSEA was performed), including core genes such as STAT2, IFNG, and IRF1, in HCT116 cells after IFN-β treatment. **B** In vivo therapeutic experiment in an orthotopic colorectal cancer mouse model. Treatment groups include Control, Trametinib monotherapy, anti-PD1 monotherapy, and Combination therapy. Representative mouse bioluminescence images (BLI) are shown on days 7, 12, 17, 22, and 27. **C** Tumor growth kinetics curve monitored by Bioluminescence Imaging (BLI). **D** Representative images of tumors at the endpoint of the orthotopic colorectal cancer mouse experiment for Control, Trametinib monotherapy, anti-PD1, and Combination therapy groups. Scale bar = 5 mm. **E** Sankey diagram and bubble plot showing KEGG pathway enrichment analysis of differentially expressed genes in mouse tumors before and after anti-PD1 treatment, highlighting changes in key pathways such as Cell Cycle and MAPK Signaling Pathway. The *P*-value from the enrichment analysis was used to determine the statistical significance of pathway enrichment. **F** GSEA analysis of in vivo samples before and after anti-PD1 treatment. The pathway "Regulation of nuclear cell cycle DNA replication" is significantly downregulated (NES = −1.56, *P* = 0.02, GSEA was performed), while the pathway "Response to interferon beta" is significantly upregulated (NES = 1.31, *P* = 0.05, GSEA was performed). **G** RT-qPCR detection of the expression levels of MAPK pathway downstream genes FOS, EGR1, and DUSP6 in HCT116 and SW620 cells under Control, IFNB, Trametinib, and Combination treatment. The *P*-value indicates the statistical difference (ANOVA). **H** Flow cytometry analysis of tumor-infiltrating lymphocytes. The scatter plot shows the proportion of CD8^+^ T cells and the expression level of PD-1 in each treatment group, with the corresponding statistical quantification presented at the bottom bar graph. The *P*-value indicates the statistical difference (ANOVA). **I** Representative image of immunofluorescence staining of tumor tissue sections (Blue: DAPI, Red: CD8, Green: TNF-alpha) and quantification of CD8^+^TNF-α^+^ double-positive cell density, showing that the combination therapy significantly increased the infiltration of cytotoxic T cells (*P* < 0.001, ANOVA was used to compare the means of cell density across the groups). IFN-β: Interferon-beta; GSEA: Gene Set Enrichment Analysis; NES: Normalized Enrichment Score; BLI: Bioluminescence Imaging; anti-PD1: Anti-Programmed Death-1 (PD-1 blockade); KEGG: Kyoto Encyclopedia of Genes and Genomes; MAPK: Mitogen-Activated Protein Kinase; RT-qPCR: Reverse Transcription quantitative Polymerase Chain Reaction; PD-1: Programmed Cell Death Protein 1; DAPI: 4',6-diamidino-2-phenylindole; TNF-α: Tumor Necrosis Factor alpha

We performed GSEA on tumor cells treated with IFN-β to identify key molecular events. Figure 8A shows that both the Negative Regulation Of Cell Cycle G1 S Phase Transition gene set (NES: 1.69; *P* < 0.001) and the Interferon Mediated Signaling Pathway gene set (NES: 2.03; *P* < 0.001) were significantly enriched on tumor cells treated with IFN-β treatment. Building upon the in vitro mechanism of IFN-β signaling inducing MEK-dependent dormancy, we investigated the in vivo efficacy of the combination of anti-PD1 and the MEK inhibitor Trametinib in the MC38 orthotopic tumor-bearing mouse model. Longitudinal BLI of tumor photon radiation (Fig. 8B) and quantitative analysis (Fig. 8C) indicated that while Trametinib or anti-PD1 monotherapy showed moderate tumor control, the combination therapy group exhibited significantly stronger and more durable tumor growth inhibition. The log BLI remained the lowest in the combination group throughout the experiment (Day7 to Day27). Representative images of tumors and intestines collected at the experimental endpoint clearly showed that the tumor burden was minimal in the combination group compared to the control or any monotherapy group, macroscopically validating the enhanced efficacy (Fig. 8D). Moreover, similar synergistic anti-tumor activity of Trametinib and anti-PD1 was observed in the MC38 model using immune-competent mouse subcutaneous colon cancer xenografts, without causing significant systemic toxicity at therapeutic doses (Fig. S11A-D).

To further decipher the cellular basis of this synergistic effect at the microscopic level, we performed multicolor immunofluorescence (mIF) to simultaneously evaluate tumor cell dormancy (p27), apoptosis (Cleaved Caspase-3, CC3), and proliferation (Ki67) within the residual tumor tissues (Figure S12).

Quantitative analysis revealed that anti-PD1 monotherapy significantly enriched the dormant subpopulation, with the proportion of p27^+^ cells rising from ~ 8% in the control group to ~ 42% (*P* = 0.0003), confirming that immune pressure indeed "pushes" a large fraction of tumor cells into a quiescent state. Crucially, while these p27^+^ cells remained viable under anti-PD1 monotherapy, the addition of Trametinib triggered a dramatic increase in apoptosis, with the percentage of CC3^+^ cells reaching ~ 33.5% (*P* < 0.001). Notably, mIF co-staining demonstrated that these apoptotic signals predominantly localized within the p27^+^ dormant "reservoir," suggesting that MEK inhibition specifically eliminates the adaptive dormant seeds induced by PD-1 blockade. Furthermore, the combination group exhibited the lowest Ki67^+^ proliferative index, indicating a robust and sustained suppression of tumor growth. These histological findings provide direct evidence for our proposed "two-step" eradication strategy: anti-PD1 first enriches a dormant population, which is subsequently eliminated through the blockade of MEK-dependent survival signaling.

Transcriptomic sequencing analysis comparing mice treated with anti-PD1 monotherapy to the control group revealed that the MAPK signaling pathway was a core regulated pathway associated with the gene expression changes induced by anti-PD1 treatment, as presented in the Sankey diagram and scatter plot (Fig. 8E). GSEA results further suggested that, compared to the control group, Response to interferon beta showed significant positive enrichment, whereas regulation of nuclear cell cycle DNA replication showed significant negative enrichment after anti-PD1 use (Fig. 8F).

Since the MAPK pathway plays a core role in cell growth and stress response, we hypothesized that Trametinib could effectively interfere with the tumor cell survival signals used to maintain or escape IFN-β-induced dormancy. To precisely capture the molecular transition of CRC cells upon IFN-β exposure, we focused on immediate early genes (IEGs), which serve as the primary genomic responders to cytokine stress [64]. Rather than selecting isolated tracking markers, we established a "Survival Triad" framework comprising DUSP6, EGR1, and FOS based on their functional complementarity and pathway coupling. Within this triad, EGR1 acts as a gatekeeper to orchestrate cell cycle exit [65, 66]; DUSP6 functions as a signaling rheostat to maintain low-level ERK homeostasis and prevent apoptotic collapse [67]; and FOS drives stress-adaptive transcriptional remodeling to anchor a long-term survival imprint [68]. Consistently, we reasoned that Trametinib targets this survival machinery by robustly inhibiting the expression of these vital IEGs. To validate this hypothesis, we used RT-qPCR to validate the effects of IFN-β and Trametinib monotherapy or combination treatment on the expression of key IEGs in two CRC cell lines (HCT116 and SW620). In both cell lines, IFN-β alone significantly enhanced the expression of the three IEGs: DUSP6, EGR1, and FOS, relative to the control group. This suggests that while IFN-β signaling inhibits the cell cycle, it might activate the MAPK pathway or bypass signals, inducing IEGs as an adaptive survival or signal compensation mechanism for the tumor cells. The IFN-β combined with Trametinib treatment group completely suppressed the expression levels of IEGs to baseline or lower levels, significantly below the peak induced by IFN-β monotherapy (Fig. 8G).

To further elucidate the immunological basis for the potent anti-tumor efficacy of the combination treatment and the mechanism by which it breaks IFN-β-induced dormancy, we subsequently evaluated the ability of Trametinib and anti-PD1 to reshape the tumor immune microenvironment. Compared to the control group (15.5%), both Trametinib (18.4%) and anti-PD1 (27.5%) monotherapies moderately increased the proportion of tumor-infiltrating CD8^+^ T cells. Crucially, the combination therapy group showed the most significant enhancement in infiltration (38.4%, significantly higher than the control, Trametinib monotherapy, and anti-PD1 monotherapy groups). This indicates a powerful immune cell recruitment or expansion effect of the combination regimen. Anti-PD1 monotherapy led to a decrease in the PD-1 expression ratio (39.2%), consistent with the flow cytometry characteristic after the blocking agent binds to the target. Notably, the combination therapy group further reduced the PD-1 expression ratio to the minimum (24.7%, significantly lower than all other groups), which may reflect the reversal of T cell exhaustion or dynamic changes in the CD8^+^ T cell population due to the MEK inhibitor (Fig. 8H).

To visually validate these findings and the impact of therapy on the tumor-immune interface, we performed multicolor immunofluorescence (mIF) staining on tumor allograft sections (Fig. S13). Consistent with our transcriptomic data, Ifnar1 (green) was found to be expressed within tumor nests, with its intensity and spatial distribution notably modulated in the combination therapy group, supporting the role of MEK inhibition in interfering with adaptive signaling plasticity (Fig. S13A). Furthermore, co-staining for CD8 (magenta) and PD1 (yellow) revealed that while anti-PD1 monotherapy increased CD8^+^ T cell recruitment, the combination therapy group exhibited a markedly higher density of CD8^+^ T cells with reduced PD1 signal intensity and a more favorable spatial distribution compared to monotherapies (Fig. 8I and Fig. S13B). This direct visualization confirms that the combination strategy not only recruits more effector cells but also creates a more receptive microenvironment by dismantling the dormant-associated immune barrier.

Immunofluorescence staining and quantitative analysis assessed T cell cytotoxic function. While monotherapies (Trametinib or anti-PD1) both mildly increased the number of functionally activated CD8^+^ T cells, the combination therapy group resulted in an explosive increase in CD8^+^TNF-α^+^ cell density. This density was significantly higher than all monotherapy and control groups (Fig. 8I). These data explicitly confirm that the combination of MEK inhibition and PD-1 blockade not only promotes the infiltration of CD8^+^ T cells but, more critically, achieves synergistic immune activation by enhancing the cytotoxic function of these infiltrating cells.

In conclusion, highly effective PD-1 immune blockade, while suppressing tumor cell proliferation via IFN-β signaling, also drives tumor cells into an adaptive dormant state. This dormancy mechanism is dependent on the MAPK pathway (MEK-dependent). The combined application of the MEK inhibitor Trametinib and anti-PD1 effectively breaks this adaptive dormancy and achieves significant and durable synergistic anti-tumor efficacy by remodeling the tumor immune microenvironment.

## Discussion

Recent advances in cancer dormancy research have redefined this cellular state: it is no longer viewed as a passive state of quiescence but rather an active, epigenetically driven phenotype [69]. This phenotype, acquired through the hallmark of phenotypic plasticity, allows cells to trade proliferative capacity for heightened heterogeneity, stemness, and invasiveness. This malignant phenotypic switching is a critical adaptive mechanism that ensures tumor survival against adverse conditions, such as therapeutic pressure or immune attack, and serves as a potential reservoir for tumor recurrence and metastasis [5, 70].

In this study, we observed that recurrent tumors exhibited a significantly higher dormancy score than primary tumors (Fig. S1B/C), an observation strongly supporting the hypothesis that surviving cancer cells retain a distinctive 'survival imprint.' This finding brings to the fore a critical biological question: do the genes driving this phenotype predominantly function as cell cycle brakes, or do they constitute a robust, general machinery deployed to counteract adverse microenvironmental pressures? Based on our functional enrichment cascades and core gene architecture, we propose that the phenotype quantified by the CADS represents an active, 'survival-centric' defense program. Although transient cell cycle arrest (evidenced by G0/G1 accumulation) is a hallmark of dormancy, mere cessation of proliferation is fundamentally insufficient for cells to survive under prolonged hypoxia, nutrient deprivation, or active immune surveillance. Intriguings, several prominent members that heavily contribute to the CADS encode membrane-anchored shields that confer immediate survival advantages. For example, CD55 acts as a pivotal complement regulatory protein that directly thwarts complement-dependent cytotoxicity (CDC) by inhibiting C3/C5 convertases [71]. Furthermore, PLAUR (uPAR) and EMP1 function as critical hubs integrating extracellular matrix cues; upon stress exposure, they switch the intracellular network from a 'proliferation-oriented' to a 'stress-adaptive' modality, activating the FAK-integrin survival axis to preclude apoptosis [72–74]. This 'survival imprint' is highly consistent with the evolving concept of Drug-Tolerant Persisters (DTPs), which exploit stable transcriptional and metabolic memory to prioritize genomic integrity and anti-apoptotic viability over growth [75, 76]. Consequently, we redefine the CADS not merely as a passive dormancy index, but as a dynamic 'defense indicator' reflecting the fitness and fitness-potential of residual tumor seeds during clinical relapse.

In this study, our integrated analysis of Figure S2 uncovers a profound correlation between the Dormancy Score and the tumor genomic landscape. In the TCGA-COAD cohort, the significant positive correlation between the DS and TMB (R = 0.27) suggests that entering dormancy represents an active evolutionary adaptation to cope with heightened immunogenicity and severe immune negative pressure [77]. Conversely, at the structural level, the negative correlation between the DS and CNV profiles demonstrates that the dormancy phenotype relies heavily on precise transcriptional reprogramming rather than gross genomic rearrangements or amplifications. This distinct 'high-TMB, low-CNV' genomic signature delineates a unique evolutionary landscape for dormant cells: it allows them to preserve long-term evolutionary adaptability while executing transient microenvironment-driven survival programs, such as the IFN-β axis, to weather external therapeutic or immune bottlenecks [68].

Cancer dormant cells safeguard themselves against cytotoxic insult by maintaining a quiescent or slow-cycling state, thereby circumventing immune surveillance [78]. Current paradigms emphasize the capacity of these cells to orchestrate an immunosuppressive niche-either through direct interference with immune effectors or via indirect modulation of the TME [59, 78, 79]. However, our deep characterization of single-cell transcriptomic landscapes reveals a strikingly distinct, immunologically active landscape. Single-cell analysis demonstrated a significant enrichment of cDC1s-pivotal for antigen cross-presentation-within the high-CADS group. Critically, GSEA confirmed the robust activation of type I interferon signaling, DC-mediated antigen processing, and T-cell-dependent anti-tumor immunity in high-CADS tumors. The concomitant elevation of IFN-β production signatures suggests that dormant cells are not merely passive evaders of immunity; rather, they serve as central orchestrators of microenvironmental remodeling via the IFN pathway. This functional reprogramming extends to the effector compartment, where T cells in the high-CADS environment exhibit heightened cytotoxicity scores. We propose that high CADS tumor cells maintain a "primed" TME by secreting or inducing type I IFN signals. Given that the high-CADS group exhibits therapeutic resistance despite this robust immune activation (as evidenced by the protective effect of IFN-β in Fig. 5D), our findings suggest that the root of resistance lies not in "immune escape," but in the adaptive survival of dormant cells in response to these very immune signals.

The cGAS-STING activation and its downstream Type I Interferon (IFN-I)/IFNAR1 signaling axis have been established as regulators of immune evasion and immunotherapy resistance [80, 81]. Activated STING signaling is known to initiate adaptive immunity. Studies have attempted to enhance IFN signals to slow tumor growth, such as combining lysine-restricted (LR) diet with anti-PD-1 therapy to mitigate the intrinsic effects of IFN-induced cancer stem cell (CSC) maintenance [82]. Similarly, high-fiber diets enhance the efficacy of ICB by modulating the microbiota to stimulate the intratumoral IFN-I-NK cell-DC axis [83]. Furthermore, our study demonstrates that the loss of Ifnar1 confers tumor sensitivity to anti-PD-1 therapy, establishing the Ifnar1 axis as a pivotal constraint on the efficacy of immune checkpoint inhibitors (ICIs). These findings underscore the strategic value of targeting the Ifnar1 axis to dismantle the survival programs inherent in the dormant phenotype. This is reinforced by the highly translational finding that IFN-β treatment actually promotes the formation of the dormant phenotype in our models, directly supporting the "IFN Signal Paradox." In dormant tumor cells, the effect of the IFN-I signal is reshaped into a survival cue rather than an apoptotic trigger. Our results challenge the canonical view that dormancy inevitably leads to an 'immunologically cold' environment. Instead, we propose a model of 'Active Immune Persistence', where high-CADS cells co-opt IFN signaling to maintain a protective dormant state despite a highly primed immune landscape. This metabolic and signaling plasticity necessitates a targeted intervention-such as MEK inhibition-to convert this paradoxical protection into a therapeutic vulnerability.

This strategy is strongly supported by the findings of Kurppa et al. [84], who demonstrated that treatment-induced dormant cells (or drug-tolerant persisters) undergo a profound transcriptional reprogramming of apoptotic pathways. Specifically, they found that while combined inhibition of EGFR and MEK drives cells into a dormant state, these cells survive by activating a YAP/TEAD-SLUG axis that suppresses BMF-mediated apoptosis. Crucially, their work establishes that the survival of these dormant populations is contingent upon the adaptive rewiring of signaling cascades, rendering them exquisitely sensitive to the dual blockade of survival programs.

Crucially, the combination of IFN-β with the MEK inhibitor Trametinib led to a significantly increased apoptosis rate. This unequivocally establishes MEK inhibition as an effective means to dismantle the survival program of dormant phenotype cells. While the MEK signaling cascade, a core component of the MAPK pathway, has long been a primary target for tumor proliferation [85, 86], recent research has increasingly focused on its role in maintaining survival in quiescent cells, with combination MEK inhibition strategies validated in models like pancreatic and non-small cell lung cancer [87, 88]. Our study is the first to explicitly define the mechanism by which MEK pathway inhibition plays a central role in cancer therapy by targeting the dormant phenotype. Specifically, the MEK inhibitor likely breaks the cells' adaptive survival response to environmental stresses (including IFN signals), thereby re-sensitizing them to cytotoxicity or immune-mediated killing. We, therefore, propose the Trametinib (MEK inhibitor) + anti-PD-1 combination strategy. By simultaneously dismantling the dormant survival program and enhancing immune cell infiltration, this therapy offers a dual-targeting approach to overcome both the MDR and immunosuppression driven by the dormant phenotype, presenting a highly translational solution to current therapeutic shortcomings.

Our study reveals that tumor cells hijack IFN-β signaling to induce dormancy and evade immune clearance, challenging the view of IFN-β as a purely antitumor factor. The balance between p38 and ERK1/2 is a key determinant of proliferation versus dormancy [76]. We found that IFN-β-induced dormancy requires sustained MEK/ERK activity to maintain survival. MEK inhibition (e.g., trametinib) not only blocks ERK-mediated survival but also preserves p38 activity, preventing dormant cells from re-entering the cell cycle and promoting apoptosis. This aligns with Sosa et al., who highlighted the p38/ERK ratio as central to the dormancy–reactivation switch [89].

### Comparison with current CRC treatments and innovation of our approach

Standard CRC treatments—fluoropyrimidines, bevacizumab, anti-EGFR agents, and immunotherapy (for MSI-H/dMMR)—face resistance and recurrence, especially in MSS patients. The BEACON CRC trial demonstrated benefits of BRAF + MEK + EGFR inhibition in BRAF V600E-mutant CRC [90], and MEK + CDK4/6 inhibition showed synergy in RAS-mutant CRC [91]. Unlike these strategies, our approach targets a therapeutic blind spot: IFN-β-induced dormant cells. Combining MEK inhibition with anti-PD-1 therapy both enhances immunotherapy and eliminates dormant cells enriched by immunotherapy.

### Strengths and limitations

The strengths of our study include mechanistic novelty by describing the IFN signaling paradox, the identification of synthetic lethal targeting of dormant cells via MEK inhibition, the demonstration of synergy between MEK inhibition and anti-PD-1 therapy, the development of the CADS biomarker for patient stratification, and multi-dimensional validation using single-cell RNA-seq, spatial transcriptomics, and in vivo models.

Several limitations should be acknowledged. The preclinical findings require clinical validation in human CRC patients. Long-term use of MEK inhibitors may cause adverse effects such as rash, diarrhea, and fatigue, necessitating dose optimization. The optimal treatment sequencing (immunotherapy first followed by MEK inhibition versus concurrent treatment) remains unknown. The CADS biomarker requires prospective validation in clinical trials. Finally, tumor heterogeneity across CRC patients may influence treatment responses.

### Future perspectives

Future work should focus on translating these preclinical findings into clinical trials of MEK inhibitor plus anti-PD-1 therapy in CRC, with particular emphasis on MSI-L/MSS patients who respond poorly to current immunotherapy. Biomarker-driven strategies based on the CADS score will be essential for patient stratification, and combination regimens incorporating standard chemotherapy such as FOLFOX should be explored. Beyond immune checkpoint blockade, the synergy of MEK inhibition with CTLA-4 inhibitors or CAR-T cell therapy warrants investigation. Deeper mechanistic studies are needed to dissect the downstream transcription factors mediating IFN-β-induced dormancy, alongside monitoring of resistance mechanisms to develop rational combination strategies. Ultimately, integrating CADS with other molecular markers into predictive models will enable personalized treatment decisions for CRC patients.

## Conclusion

In summary, this study introduces the first CADS, providing a crucial biomarker for the precise identification of patients at risk due to high-dormancy status. Our work clearly establishes the central role of the malignant dormant phenotype in CRC therapeutic resistance, offering a new perspective for dissecting resistance to both chemotherapy and ICIs. Our findings strongly support the principle that targeting the dormant phenotype is an essential new strategy for overcoming therapeutic resistance. Future research, guided by our discovered "IFN Signal Paradox" and the MEK pathway mechanism, should focus on how dormant cells utilize epigenetic or post-translational modifications to reshape the IFN signaling downstream, thereby enhancing their survival and DC suppression capabilities. Ultimately, the use of precision targeting strategies, such as MEK inhibition, promises the complete eradication of malignant dormant cells, resolving the persistent clinical challenge of tumor recurrence and metastasis.

## Supplementary Information

Supplementary Material 1. 

Supplementary Material 2.

Supplementary Material 3.

## Data Availability

The sequencing data of this study (including scRNA-seq data of mice tumor model and bulk RNA-seq data) that support the findings of this study have been deposited into CNSA with accession number CNP0008753. These data will be publicly available from the date of publication.

## Abbreviations

ANOVA: Analysis of Variance
APC: Antigen-Presenting Cell
ARRIVE: Animal Research: Reporting of In Vivo Experiments
ATCC: American Type Culture Collection
AUC: Area Under the Curve
BLI: Bioluminescence Imaging
BRCA: Breast Invasive Carcinoma
CADS: COAD-specific Dormancy Score
CCLE: Cancer Cell Line Encyclopedia
cDC1: Conventional Type 1 Dendritic Cell
cDC2: Conventional Type 2 Dendritic Cell
CNA: Copy Number Alteration
CNV: Copy Number Variation
COAD: Colon Adenocarcinoma
CRC: Colorectal Cancer
CRISPR: Clustered Regularly Interspaced Short Palindromic Repeats
CSC: Cancer Stem Cell
CTRP: Cancer Therapeutics Response Portal
DC: Dendritic Cell
DMEM: Dulbecco's Modified Eagle Medium
DMSO: Dimethyl Sulfoxide
DNA: Deoxyribonucleic Acid
DTP: Drug-Tolerant Persister
DS: Dormancy Score
EDU: 5-Ethynyl-2'-deoxyuridine
EGF: Epidermal Growth Factor
EGR1: Early Growth Response 1
ELISA: Enzyme-Linked Immunosorbent Assay
EMT: Epithelial-Mesenchymal Transition
EPO: Erythropoietin
EPOR: Erythropoietin Receptor
ERK: Extracellular Signal-Regulated Kinase
FACS: Fluorescence-Activated Cell Sorting
FBS: Fetal Bovine Serum
FOS: FBJ murine osteosarcoma viral oncogene homolog
FPKM: Fragments Per Kilobase of transcript per Million mapped reads
GDSC: Genomics of Drug Sensitivity in Cancer
GEO: Gene Expression Omnibus
GFP: Green Fluorescent Protein
GSEA: Gene Set Enrichment Analysis
GSVA: Gene Set Variation Analysis
HCC: Hepatocellular Carcinoma
HNSCC: Head and Neck Squamous Cell Carcinoma
HNSC: Head and Neck Squamous Cell Carcinoma
HVG: Highly Variable Gene
ICB: Immune Checkpoint Blockade
ICIs: Immune Checkpoint Inhibitors
IF: Immunofluorescence
IFN-β: Interferon-beta
Ifnar1: Interferon Alpha and Beta Receptor Subunit 1
IFN-I: Type I Interferon
IEG: Immediate Early Gene
i.p.: Intraperitoneal
ITH: Intratumoral Heterogeneity
IVIS: In Vivo Imaging System
KEGG: Kyoto Encyclopedia of Genes and Genomes
LUAD: Lung Adenocarcinoma
MAPK: Mitogen-Activated Protein Kinase
MEK: MAPK/ERK Kinase
MHC: Major Histocompatibility Complex
MMRd: Mismatch Repair-deficient
MMRp: Mismatch Repair-proficient
mregDC: Mature Regulatory DC
MSI-H: Microsatellite Instability-High
MSI-L: Microsatellite Instability-Low
MSS: Microsatellite Stable
NES: Normalized Enrichment Score
NMF: Non-Negative Matrix Factorization
OS: Overall Survival
OV: Ovarian Serous Cystadenocarcinoma
PCA: Principal Component Analysis
PD-1: Programmed Cell Death Protein 1
pDC: Plasmacytoid Dendritic Cell
PI: Propidium Iodide
PRAD: Prostate Adenocarcinoma
QC: Quality Control
qPCR: Quantitative Polymerase Chain Reaction
READ: Rectal Adenocarcinoma
RFS: Recurrence-Free Survival
RNA: Ribonucleic Acid
RNA-seq: RNA Sequencing
ROC: Receiver Operating Characteristic
RT-qPCR: Reverse Transcription Quantitative PCR
SA-β-gal: Senescence-Associated β-galactosidase
SARC: Sarcoma
SCNV: Somatic Copy Number Variation
scRNA-seq: Single-Cell RNA Sequencing
SEM: Standard Error of the Mean
ssGSEA: Single-Sample Gene Set Enrichment Analysis
STAD: Stomach Adenocarcinoma
TAM: Tumor-Associated Macrophage
TCGA: The Cancer Genome Atlas
Tcm: Central Memory T Cell
TDLN: Tumor-Draining Lymph Node
TIDE: Tumor Immune Dysfunction and Exclusion
TIL: Tumor-Infiltrating Lymphocyte
TMB: Tumor Mutation Burden
TME: Tumor Microenvironment
TNF-α: Tumor Necrosis Factor-alpha
UMAP: Uniform Manifold Approximation and Projection
UMI: Unique Molecular Identifier
