# Supplementary material for: From paradox to target: IFN-β hijacks MEK signaling to drive a cell death-evading dormant phenotype in colorectal cancer
Source: J Exp Clin Cancer Res. 2026 Jun 27;45:194. doi: 10.1186/s13046-026-03768-6 (PMC13576298; doi:10.1186/s13046-026-03768-6)
Supplement: Supplementary file 1 [file 13046_2026_3768_MOESM3_ESM.docx]

Figure S1: Functional Annotation, Immune Features, and Clinical Relevance of the Dormancy Signature

(A) Enrichment map illustrating the functional annotation of the 224 dormancy-associated genes (intersecting Ren_Dormant_UP and Weng_Dormant_UP) across KEGG pathways and GO terms (MF/CC/BP).

(B) Comparison of the Dormancy Score (DS) distribution between adjacent non-tumor tissues and tumor tissues in the TCGA pan-cancer cohort. The P-value indicates the statistical difference (Student's t-test).

(C) Distribution of the DS in recurrent versus non-recurrent samples within the TCGA pan-cancer cohort. The P-value indicates the statistical difference (Student's t-test).

DS: Dormancy Score; UP: Upregulated; KEGG: Kyoto Encyclopedia of Genes and Genomes; GO: Gene Ontology; MF: Molecular Function; CC: Cellular Component; BP: Biological Process; TCGA: The Cancer Genome Atlas

Figure S2: Correlation of the Dormancy Score with Tumor Genomic Alterations

(A) Correlation analysis of the Dormancy Score (DS) with Tumor Mutation Burden (TMB). The x-axis represents the regression coefficient, and the y-axis represents -log_10(P-value). The P-value indicates the significance of the correlation (Pearson's correlation).

(B) Scatter plot showing the correlation between the DS and TMB in Colon Adenocarcinoma (COAD) samples. The P-value indicates the significance of the linear relationship (Pearson's correlation).

(C) Pan-cancer correlation analysis between the DS and the Copy Number Variation (CNV) score. The x-axis represents the regression coefficient, and the y-axis represents -log_10(P-value). The P-value indicates the significance of the correlation (Pearson's correlation).

(D) Correlation of the DS with genomic alterations at different levels, including focal level and chromosome arm level. The P-value indicates the significance of the correlation (Pearson's correlation).

DS: Dormancy Score; TMB: Tumor Mutation Burden; COAD: Colon Adenocarcinoma; CNV: Copy Number Variation

Figure S3: Association of the Dormancy Score with Chemosensitivity and Immunotherapy Response Predictors

(A) Comparison of sensitivity to 14 chemotherapeutic drugs (e.g., Capecitabine, Docetaxel) between the low-DS and high-DS groups. The x-axis represents the group, and the y-axis represents the AUC value (lower AUC indicates higher sensitivity). The P-value indicates the statistical difference in AUC between the groups (Student's t-test).

(B) Association between the Dormancy Score (DS) and the Tide score in different published cohorts (e.g., Cho et al., 2020).

DS: Dormancy Score; AUC: Area Under the Curve; Tide: Tumor Immune Dysfunction and Exclusion

Figure S4: Single-Cell Characterization and Copy Number Variation Analysis of Tumor and Non-Malignant Cells

(A-C) UMAP clustering analysis of the colorectal cancer single-cell datasets GSE178341 (A), LEE (B), and GSE166555 (C), illustrating the distribution of various cell types, including stromal cells, myeloid cells, and endothelial cells.

(D) Heatmap of Chromosomal Copy Number Alterations (CNA) for non-malignant (normal tissue reference) and malignant cells from the GSE178341 dataset. The x-axis represents the chromosome number, and the y-axis represents patient samples and cell clusters. Reference (normal tissue) and Non-malignant cells showed no significant CNA patterns across all chromosomes, with log2-ratio close to 0 (white/light colors). In contrast, many cells from patients exhibited extensive CNA across multiple chromosomes. Copy number gain (red): Amplifications (log2-ratio> 0) were observed in many patient cells (e.g., chromosomes 1, 3, 8, 12, 19, and 20), with log2-ratio of 1 indicated by dark red. Copy number loss (blue): Deletion regions (log2-ratio < 0) were visible on certain chromosomes (e.g., chromosomes 4, 10, and 17), with log2-ratio of -1 indicated by dark blue.

UMAP: Uniform Manifold Approximation and Projection; CNA: Copy Number Alterations

Figure S5. Correlation of the CADS with chemotherapy resistance, epithelial-mesenchymal transition, and intratumoral heterogeneity.

(A, B) Scatter plots showing the correlation between the mean COAD-specific Dormancy Score (mean_CADS) and key biological features in the LEE cohort (A) and the GSE166555 cohort (B). Plots illustrate the relationship of CADS with intratumoral heterogeneity (ITH_euc, left), the epithelial-specific high-risk gene set signature (mean_epiHR, center), and the chemotherapy resistance score (mean_chemoresistance, right). Pearson's correlation coefficient (R) and the corresponding P-value are annotated for each comparison.

CADS, COAD-specific Dormancy Score; ITH_euc, Intratumoral Heterogeneity (Euclidean distance); epiHR, epithelial-specific high-risk gene set; COAD, Colon Adenocarcinoma.

Figure S6: Cell Cycle State and Molecular Signatures of Malignant Cells based on the CADS

(A-B) Cell cycle state inference and CADS validation in malignant cells. The tricycle method was used to calculate the cell cycle score, classifying cells into Quiescent or Actively proliferating states. The CADS distribution was compared between Quiescent and Actively proliferating cells in the LEE (A), and GSE166555 (B) datasets. The P-value confirms that Quiescent cells exhibit a significantly higher CADS than actively proliferating cells (Student's t-test).

(C-D) Differential expression analysis of proliferation and dormancy markers based on CADS. Comparison of gene expression between the top 25% of malignant cells (High-CADS) and the bottom 25% of malignant cells (Low-CADS) based on CADS. The P-value indicates the statistical difference in gene expression (Student's t-test).

CADS, COAD-specific Dormancy Score.

Figure S7 Microsatellite instability and mismatch repair status are associated with higher CADS in colorectal cancer.

(A) Comparison of mean CADS between tumors with microsatellite instability-low/stable (MSI-L/MSS) and microsatellite instability-high (MSI-H) status in the TCGA-COAD cohort. The box plot shows the score distribution across different microsatellite stability states. Results indicate that MSI-H tumors have a significantly higher CADS than MSI-L/MSS tumors (P < 0.05, Student's t-test).

(B) Quantitative comparison of mean CADS between mismatch repair-proficient (MMRp) and mismatch repair-deficient (MMRd) tumors in the GSE39582 cohort. The box plot results demonstrate that MMRd tumors exhibit a significantly higher dormancy score than MMRp tumors (P < 0.05, Student's t-test).

CADS, COAD-specific Dormancy Score; COAD, colon adenocarcinoma; MMR, mismatch repair; MSI, microsatellite instability; TCGA, The Cancer Genome Atlas.

Figure S8. Characterization of dendritic cell subsets and CD8⁺ T cell functional states associated with CADS.

(A) Dot plot depicting the expression patterns of canonical marker genes across the four identified dendritic cell (DC) subsets: regulatory DCs (mregDCs), conventional DC type 1 (cDC1), conventional DC type 2 (cDC2), and plasmacytoid DCs (pDCs). Dot size represents the percentage of cells expressing the gene, and color intensity represents the average expression level.

(B–D) Quantitative comparison of DC subset proportions between the CADS-high and CADS-low groups. Box plots show the relative fractions of cDC2 (B), pDC (C), and mregDC (D).

(E) Spatial transcriptomics data were normalized by library size and log-transformed. Gene set variation analysis (GSVA) was performed to compute enrichment scores for three signatures: cDC1 (conventional type 1 dendritic cells), CADS (cancer dormancy-associated signature), and IFN-β signaling. Each panel represents a distinct tissue region from two patients (6723_KL and 7003_AS). Scores are color-coded and projected onto the corresponding spatial coordinates to visualize local enrichment patterns. Scale bars represent signature scores, with higher values indicating greater enrichment.

(F) UMAP visualization of tumor-infiltrating CD8⁺ T cells. Cells were partitioned into seven functional subsets based on transcriptional profiles: effector (CD8_eff), exhausted (CD8_ex), naïve (CD8_naive), central memory (CD8_Tcm), effector memory (CD8_Tem), tissue-resident memory (CD8_Trm), and proliferating (CD8T_proliferating).

(G) Dot plot showing the expression of key lineage-defining and functional genes that characterize the CD8⁺ T cell subsets defined in (E), including SELL, CCR7, GZMB, LAG3, and MKI67.

(H, I) Comparison of the relative abundances of CD8⁺ T cell subsets between CADS-high and CADS-low patients. Bar plots display the proportions of effector T cells (Teff) (G) and central memory T cells (Tcm) (H). The CADS-low group exhibits a significantly higher Tcm proportion (P = 0.002, Student's t-test).

(J) Gene Set Enrichment Analysis (GSEA) of biological processes enriched in CADS-high patients. The bubble plot displays significantly enriched pathways, including T cell activation, lymphocyte-mediated immunity, and response to interferon-beta. Color represents -log₁₀(P-value), and the x-axis represents the Normalized Enrichment Score (NES).

(K, L) Violin plots comparing functional signature scores between CADS-high and CADS-low groups, including the naïve/memory T cell score (J) and the T cell cytotoxicity score (K).

CADS, COAD-specific Dormancy Score; DC, dendritic cell; mregDC, regulatory DC; cDC1/cDC2, conventional DC type 1/2; pDC, plasmacytoid DC; UMAP, uniform manifold approximation and projection; GSEA, gene set enrichment analysis; NES, normalized enrichment score; Teff, effector T cell; Tcm, central memory T cell.

Figure S9. Dynamic remodeling of tumor-infiltrating immune cell landscape upon anti-PD-1 treatment.

(A) Dot plot showing the expression percentage (dot size) and mean expression level (color intensity) of key marker genes across major tumor-infiltrating immune cell subsets, including regulatory T cells (Tregs), natural killer cells (NK), B cells, monocytes/macrophages (Mo/Mφ), dendritic cells (DC), and neutrophils.

(B) UMAP dimensionality reduction analysis illustrating the global composition of CD45⁺ immune cells before and after anti-PD-1 treatment.

(C) Bar chart comparing the relative proportions of major immune cell subsets between the control (Ctrl) and anti-PD-1 treated groups. The accompanying line graph plots the log₂ fold change (log₂FC) for each subset.

(D) Proportional changes of dendritic cell (DC) and CD8⁺ T cell populations within total CD45⁺ cells before and after anti-PD-1 treatment.

(E) Two-dimensional density plots comparing the T cell fitness score and exhaustion score of CD8⁺ T cells before and after anti-PD-1 treatment.

anti-PD-1, anti-programmed cell death protein 1; Treg, regulatory T cell; NK, natural killer cell; Mo/Mφ, monocyte/macrophage; DC, dendritic cell; UMAP, uniform manifold approximation and projection; log₂FC, log₂ fold change.

Figure S10. IFN-β sensitizes SW620 colorectal cancer cells to MEK inhibition

A. Flow cytometric analysis of apoptosis using Annexin V/PI staining in SW620 cells treated with IFN-β (5 ng/ml) alone or in combination with Oxaliplatin (10 uM). The bar graph (right) represents the percentage of apoptotic cells, with P-values indicating statistical significance (Student's t-test).

B. Flow cytometric analysis (Annexin V/PI double staining) detecting the apoptotic effect of IFN-beta combined with Selumetinib (0.5 uM) or Trametinib (0.5 uM) treatment on SW620 cells. The lower bar graph shows the percentage of apoptosis, with P-values indicating the significance of the synergistic or additive effects (Student's t-test).

Figure S11: Synergistic Anti-tumor Effect of Trametinib and anti-PD1 Combination Therapy in the MC38 Colorectal Cancer Model

(A) Tumor volume change over time in MC38 tumor-bearing mice under different treatment regimens. Combination therapy (Trametinib + anti-PD1) demonstrates the strongest inhibition of tumor growth, significantly superior to monotherapy and control groups. The P-value indicates the statistical difference (Two-way ANOVA with repeated measures).

(B) Representative photographs of excised tumors from each treatment group at the end of treatment (Day 14).

(C) Mouse body weight (g) change over time (days) during the treatment period. Body weight remained stable across all treatment groups, with no statistically significant difference compared to the control group (ns, not significant; Student's t-test), indicating good tolerance of the therapeutic regimens.

(D) Quantitative analysis of the mean tumor weight (mg) for each group at the end of treatment (Day 14). Tumor weight in the combination therapy group was significantly lower than all other groups (p < 0.001, Student's t-test), supporting the synergistic anti-tumor effect shown by the tumor volume data.

Annotation of statistical significance. *** indicates p < 0.001. ns indicates no significant difference. anti-PD1: Anti-Programmed Death-1 (PD-1 blockade); ANOVA: Analysis of Variance; ns: Not Significant

FigureS12. Impact of Combination Therapy on Tumor Cell Dormancy, Apoptosis, and Proliferation

(A) Immunofluorescence (IF) Staining Results
Representative multicolor immunofluorescence images of tumor tissues from orthotopic colorectal cancer mouse models across different treatment groups: Control, anti-PD1, Trametinib, and Combination therapy. The staining panels include: p27 (green, marker for dormant/quiescent cells), Cleaved Caspase-3 (red, marker for cell apoptosis), Ki67 (magenta, marker for cell proliferation), and DAPI (blue, nuclear counterstain). "Merge" represents the overlay of all four channels. Scale bar: 200 μm.

(B) Quantitative Analysis

Left: Percentage of p27-positive cells. The anti-PD1 group shows a significantly higher proportion of p27^+^ cells compared to the Control group (P=0.0003), confirming that anti-PD1 treatment enriches the dormant cell subpopulation.

Middle: Percentage of Cleaved Caspase-3-positive cells. The Combination group exhibits significantly higher apoptosis levels than the anti-PD1 monotherapy group (P=0.0007), indicating that Trametinib specifically triggers apoptosis in the pre-enriched dormant cells.

Right: Percentage of Ki67-positive cells. The Combination group shows a significant reduction in proliferating cells compared to the Control group (P=0.0018), demonstrating that the synergistic effect of Trametinib and anti-PD1 effectively suppresses overall tumor cell proliferation.

Figure S13. Spatial validation of Ifnar1 signaling and T-cell dynamics in tumor allografts upon combination therapy. (A) Multicolor immunofluorescence (mIF) analysis of Ifnar1 expression. Representative images showing the protein-level expression and spatial distribution of Ifnar1 (green) in tumor nests across the indicated treatment groups (Control, Trametinib, Anti-PD1, and Combination). DAPI (blue) was used as a nuclear counterstain. Note the modulation of Ifnar1 intensity and spatial patterning specifically in the combination therapy group, suggesting a remodeling of adaptive signaling plasticity in response to combined MEK inhibition and PD-1 blockade. Scale bar: 50 μm. (B) mIF visualization of T-cell infiltration and exhaustion status. Representative images illustrating the spatial distribution of cytotoxic T cells (CD8^+^, magenta) and the expression of the exhaustion marker PD1 (yellow) within the tumor microenvironment. The combination group demonstrates a synergistic increase in CD8+ T-cell density with a markedly improved spatial distribution and altered PD1 expression profile. These findings support the potentiation of anti-tumor immunity through the disruption of the dormant-associated immune barrier. Scale bar: 50 μm.

Supplementary Table 1. Comprehensive gene lists defining the 'dormant_up' and 'reactive_up' subpopulations.

Supplementary Table 2. Detailed information on reagents, antibodies, and equipment.
